# Cellulose Structures as a Support or Template for Inorganic Nanostructures and Their Assemblies

**DOI:** 10.3390/nano12111837

**Published:** 2022-05-27

**Authors:** Alojz Anžlovar, Ema Žagar

**Affiliations:** National Institute of Chemistry, Hajdrihova 19, SI-1000 Ljubljana, Slovenia; ema.zagar@ki.si

**Keywords:** cellulose, metallic and metal oxide nanostructures, metal sulfide nanostructures, mesoporous nanostructures, hierarchical nanostructures, heterogeneous catalysis

## Abstract

Cellulose is the most abundant natural polymer and deserves the special attention of the scientific community because it represents a sustainable source of carbon and plays an important role as a sustainable energent for replacing crude oil, coal, and natural gas in the future. Intense research and studies over the past few decades on cellulose structures have mainly focused on cellulose as a biomass for exploitation as an alternative energent or as a reinforcing material in polymer matrices. However, studies on cellulose structures have revealed more diverse potential applications by exploiting the functionalities of cellulose such as biomedical materials, biomimetic optical materials, bio-inspired mechanically adaptive materials, selective nanostructured membranes, and as a growth template for inorganic nanostructures. This article comprehensively reviews the potential of cellulose structures as a support, biotemplate, and growing vector in the formation of various complex hybrid hierarchical inorganic nanostructures with a wide scope of applications. We focus on the preparation of inorganic nanostructures by exploiting the unique properties and performances of cellulose structures. The advantages, physicochemical properties, and chemical modifications of the cellulose structures are comparatively discussed from the aspect of materials development and processing. Finally, the perspective and potential applications of cellulose-based bioinspired hierarchical functional nanomaterials in the future are outlined.

## 1. Introduction and Scope

With the aim to meet the global challenges that humanity is facing nowadays such as a lack of energy and fossil resources as well as increasing environmental issues, green and progressive strategies are expected to sustain the research impulse. In this context, cellulose, with its derivatives, will certainly play a significant role in this demanding task since it has an annual average output of 10^11^–10^12^ tons in nature and only 2 × 10^6^ tons are used by people [1,2]. Bio-based materials are attractive as the basis for sustainable development and innovative solutions to a wide range of technological challenges as well as environmental problems. Cellulose and cellulose-based materials are not only earth abundant and biocompatible, but also contain intrinsic structures for transformative device performance [3]. While many innovative structures and devices as well as applications have been published and demonstrated, substantial challenges still exist in the fundamental science, and the commercialization of cellulose-based materials is a necessity in solving the emerging problems and demands [3,4,5,6]. The great advantage of cellulose or cellulose-based products and materials is that they can be produced from various byproducts or waste products emerging during food production such as various straws, peelings, husks, bagasse, corn stalks, or cobs, etc., or during wood processing [7,8,9,10].

Cellulose is the most abundant natural polymer globally, and is present in a large number of living species, mostly plants and bacteria, but also in animals [11,12]. It is a biodegradable and biocompatible as well as renewable polymer and it is a green alternative to fossil-fuel carbon sources [13,14]. Cellulose has also attracted the special attention of the scientific community as a versatile and abundant “green” template [15]. The formation of functional nanomaterials via biomineralization or biotemplating is a topic that is attracting an enormous amount of interest from academia and the industry because it is promising for a more precise control over the positioning and connecting of different functional nanostructures into complex nano- and macro-devices [16,17].

The natural cellulose molecule is actually a long polysaccharide chain. It consists of a variable number of β-1,4 linked glucopyranose rings as repeating units [18]. Cellulosic polymer chains are, due to a vast number of hydroxyl groups, associated via hydrogen bonds forming bundles of fibrils (microfibrillar aggregates), where highly ordered regions (crystalline domains) alternate with disordered regions (amorphous domains) inside the cellulose fibrils, which constitute the walls of plant cells [19,20]. This highly ordered structure gives cellulose a very high elastic modulus and specific strength, making it an ideal reinforcing material for polymer matrices [21,22,23] in a variety of forms such as macroscopic fibers (hemp, jute, and kenaf), microfibrillated cellulose (MFC), cellulose nanofibrils (CNFs), or cellulose nanocrystals (CNCs) [13,24].

On the other hand, the scientific and research communities have experienced an immense rise in interest in the areas of nanoscience and nanotechnology during the last few decades. These are nowadays at the center of our technological progress due to their outstanding capability to manipulate matter on a nanometer scale. The capability to directly design, synthesize, and control systems at the same scale as nature is a huge scientific and technological challenge [25]. Over billions of years, nature has evolved nanoscale biological systems for the efficient production of energy and materials. By mimicking such systems, scientists aim to reach the goal of a sustainable society in the future [25]. Therefore, nanomaterials and nanotechnology have been regarded as a huge leap toward miniaturization and nanoscaling, generating various subfields with the aim to study these materials in detail. Nanotechnology as a scientific field is highly multidisciplinary; contributions from biologists, chemists, physicists, and engineers are indispensable to the advancement in the understanding the formation, application, and impact of new nanostructures and innovative nanotechnologies, which can potentially provide a very efficient approach to the production of chemicals, fuels, energy, and energy efficient materials [25,26,27,28].

### 1.1. Types and Forms of Cellulose

#### 1.1.1. Cellulose Fibers and Fibrils (CF)

Native cellulose, defined as the cellulose I polymorph, does not exist as an isolated individual molecule, but is found as highly organized assemblies of individual cellulose chains, forming extended structures-fibers. The ultrastructure of cellulose fibers exhibits a hierarchical arrangement, starting from cellulose molecules to elementary fibrils and further to micro- as well as macrofibers. When cellulose molecules are synthesized as individual molecules, they immediately undergo spinning in a hierarchical order at the site of biosynthesis. Typically, up to 36 individual cellulose molecules are self-organized into larger units known as elementary fibrils (protofibrils) that are further packed into so-called microfibrils, and these are finally assembled into cellulose fibers with diameters of a few micrometers and lengths of up to a few thousands millimeters. Depending on the biosynthesis conditions, celluloses from different sources may occur in different packing that results in the different morphologies and properties of cellulose fibers [11,20]. For various purposes, cellulose needs to be isolated from its source, followed by purification and possible modification. Methods of extraction, purification, and modification affect the physical and chemical properties of cellulose such as the chain lengths and their distribution, the degree of crystallinity, the mechanical properties, thermal stability, solubility, the distribution of functional groups in the monomer units of the cellulose polymer chain, and the ratio of inter- and intra-molecular hydrogen bonds in the cellulose molecules. These physico-chemical properties play a key role in the determination of industrial and commercial applications of cellulose fibers [29,30,31].

#### 1.1.2. Regenerated Cellulose (RC)

Regenerated cellulose, defined as a cellulose II polymorph, is obtained by the chemical regeneration process comprising the dissolution or swelling of native cellulose I fibers, followed by its reprecipitation in water. Suitable solvents for cellulose include cupric hydroxide in aqueous ammonia (cuoxam) or cupriethylenediamine (cuen), ammonia or amine/thiocyanate, hydrazine/thiocyanate, lithium chloride/N,N-dimethylacetamide (LiCl/DMAc), and N-methylmorpholine-N-oxide (NMMO)/water systems, and lately, ionic liquids (IL). Most of these solvents are limited on a pilot-scale except for NMMO, which is commercially used for the production of regenerated cellulose fibers called Lyocell [32]. Although viscose technology is still the most widely used process to manufacture regenerated cellulose fibers, this technology has many disadvantages related to environmental issues. A special case is mercerization (i.e., the swelling of the native cellulose in concentrated sodium hydroxide solution followed by the removal of the swelling agent). All of these processes finally produce fibers of the cellulose II polymorph. During the conversion (I to II), the conformation of hydroxyl groups is changed, causing a rearrangement in the entire hydrogen-bonded network while the dimensions of regenerated fibers remain mostly unchanged. Compared to cellulose I with a parallel up arrangement, the chains in cellulose II are in an antiparallel arrangement, producing a more stable fibrillary structure that is preferable for various practical applications [11,20]. One of the easiest ways to prepare regenerated cellulose is by dissolving raw cellulose in a NaOH/urea or thiourea aqueous solution [33]. An innovative approach toward regenerated cellulose is the dissolution of raw cellulose in various ionic liquids, representing a more sustainable strategy. Here, the choice of cation and anion is critical not only to the degree of the dissolution, but also to the ultimate sustainability of such processes. Studies have revealed that the interactions between the anion and cellulose play a key role in cellulose solvation, however, opinions on the cation role are still conflicting [34]. Oxidized regenerated cellulose (ORC), obtained by transforming its primary alcohol groups into carboxyl groups, is of special interest because of the various attractive medical applications and is among one of the most widespread hemostatics being used in almost any kind of surgery [2].

#### 1.1.3. Microcrystalline Cellulose (MCC)

Microcrystalline cellulose is a pure, partially degraded native cellulose I, derived from high-quality wood pulps. It is prepared either by enzyme-mediated reactive extrusion, steam explosion, or acid hydrolysis with strong mineral acids (H_2_SO_4_, HCl, or HBr). During this treatment, a partial degradation of cellulose fibers (preferentially in amorphous regions) takes place, producing a stable, chemically inactive, and physiologically inert cellulose material with attractive binding properties. The average dimension of MCC particles is above 5 µm. MCC offers the opportunity for a variety of applications (e.g., in the pharmaceutical industry as a tablet binder, in food applications as a texturizing agent, fat replacer, emulsifier, and bulking agent as well as an additive in paper and composite applications) [11]. MCC is one of the most useful and attractive fillers because of its high dilution potential, excellent compactibility at low pressures, and superior disintegration properties. Its chemical inertness and compatibility with a large number of drugs make MCC a highly attractive pharmaceutical agent [29]. Recently, various alternative sources of MCC have been studied. Although all microcrystalline cellulose is made of the same biopolymer, different raw materials can be used to obtain MCC tailored to specific needs [35].

#### 1.1.4. Microfibrillated Cellulose (MFC)

Microfibrillated (MFC) and/or nanofibrillated cellulose (NFC) consist of long, entangled, and flexible cellulose (nano)fibers composed of alternating crystalline and amorphous phases. The difference between MFC and NFC is in the average width, which is much smaller in NFC (10–50 nm) than in MFC (10–500 nm). An aqueous MFC or NFC suspension consists of interconnected hydrophilic cellulose I (nano)fibers, inducing a gelation of the suspension at low mass contents of only a few percent. These can be isolated using different mechanical treatments, which usually involve a refining step followed by a high-pressure homogenization step, although cryocrushing and grinding methods have also been applied. Mechanical treatments produce a network of interconnected cellulose microfibrils with diameters from 10 to 500 nm and aspect ratios from 50 to 100. MFC and NFC are generally produced from wood pulp, though other cellulose sources originating from agricultural byproducts are also important such as wheat straw, sugar beet pulp, oil palm kernel, potato pulp, or sugar cane bagasse, etc. [13,36]. NFC production can be commercially competitive through the choice of less energy intensive processes and by using low-cost raw materials [37,38]. Additionally, to enhance NFC production and reduce energy consumption, various physical, chemical, and biological pretreatments have been applied. NFC is frequently produced through alkali and enzymatic pretreatment followed by grinding or by using TEMPO-mediated oxidation, followed by homogenization. These pretreatments substantially improve the production yield and properties of NFC. Therefore, NFC production is a combination of several operations, and by changing their sequence, different types of NFC can be isolated [39].

#### 1.1.5. Bacterial Nanocellulose (BNC)

Cellulose fibers are secreted extracellularly by several bacterial species including Gram-negative bacteria such as *Acetobacter*, *Alcaligenes, Azotobacter*, *Rhizobium*, *Salmonella*, *Pseudomonas*, and Gram-positive bacteria such as *Sarcina ventriculi*. Bacterial cellulose (BNC) is produced most efficiently by the bacteria of the *Acetobacter* genus such as *Acetobacter G. xylinus*, *Acetobacter hansenii*, or *Aacetobacter pasteurianus* species during cultivation in an aqueous culture media containing carbon and nitrogen sources in a time of a few days. The resulting cellulosic structure is in the form of a pellicle of randomly assembled ribbon shaped fibrils with a width of less than 100 nm, which are further composed of bundles of much finer nanofibrils (2 to 4 nm in diameter). These bundles are relatively straight and dimensionally uniform, and they further form a continuous three-dimensional network. Compared to plant cellulose, BNC is chemically a cellulose of high purity without the presence of hemicellulose and lignin. It shows higher hydrophilicity and water holding capacity, and in general, a higher tensile strength, resulting from a higher degree of polymerization and ultrafine network architecture [20,40]. The microbial process is an environmentally-friendly and effective method to produce high-yield BNC from various sources. However, the physiochemical characteristics (crystallinity and morphology) as well as yield of BNC are significantly influenced by the culture medium used [39].

#### 1.1.6. Cellulose Nanocrystals (CNCs)

CNCs, also called cellulose whiskers or nanocrystalline cellulose (NCC), are predominantly isolated from cellulosic fibers by a simple process involving the acid hydrolysis of the biomass using mostly concentrated H_2_SO_4_ or other strong acids. CNCs, isolated by using (60–64%) H_2_SO_4_, possess a higher colloidal stability than CNCs produced by other acids. Severe environmental pollution concerns due to strong acid hydrolysis has encouraged the use of recyclable acids (maleic acid, formic acid, citric acid), thus ensuring a more environmentally-friendly process design. Additionally, multiple acid blends (strong and weak acid) have also been employed, resulting in highly crystalline CNCs with a better surface chemistry due to the strong and weak acid contribution. The production of CNCs can also be achieved by using oxidizing agents, ionic liquids, and subcritical water. CNCs produced by oxidation comparatively possess higher colloidal stability, crystallinity, and uniform nanoscale dimension [39]. During CNC processing, disordered (amorphous) regions of cellulose are selectively degraded, while leaving the more acid resistant crystalline regions mostly intact to produce rod-shaped cellulose nanocrystalline particles [41,42,43]. Their dimensions depend on the cellulose source and type of the isolation process used. Consequently, their widths and lengths can vary from 5 to 20 nm and from 100 nm to 1–2 μm, respectively [44]. In polar solvents, CNCs do not flocculate due to electrostatic repulsion forces originating from their negative charges on the surface, which results in stable suspensions for several months. One of the most interesting features originates from the ability of these suspensions to self-organize into stable, chiral nematic phases (characteristic for liquid crystals) that give the CNC suspension unique optical properties when a critical CNC concentration is reached [13]. The CNCs isolated by phosphoric acid (PCNCs) showed excellent flame-retardant properties due to the ability of the phosphate groups to enhance char formation, thus making PCNCs a self-extinguishing material. The incorporation of PCNCs in the nanocomposite foam also substantially improved the mechanical properties. Furthermore, PCNCs are also promising biomedical materials that can be used as a bone-scaffolding material [13,45,46]. Aside from weak acids, ionic liquids (ILs) are also used to isolate CNCs as well as other types of nanocellulose. ILs can be used alone or in combination with mineral acids, enzymatic treatment, or with physical treatment. The most suitable ILs are [BMIm]HSO_4_ and [BMIm]Cl [13,47,48]. The production of nanocellulose using ionic liquids has many benefits including the usage of atmospheric pressure, small amounts of solvents, the regeneration of ionic liquids, and working with an odorless and relatively safe solvent. On the other hand, this method also has disadvantages that include the relatively high costs of ionic liquids and the unsatisfactory efficiency of the extraction process [49].

### 1.2. Cellulose Gels as Supports or Templates for Nanostructured Materials

#### 1.2.1. Cellulose Hydrogels

Cellulose hydrogels are physically or chemically cross-linked three dimensional hydrophilic cellulose networks capable of absorbing large amounts of water (or biological fluids) and swelling. The hydrogel stability is ensured either by physical interactions (chain entanglements, van der Waals forces, hydrogen bonds, crystallite association, and/or ionic interactions) or chemical cross-links (covalent bonding) [50]. Aqueous CNC or MFC suspensions are generally characterized by their gel like properties due to the presence of long interconnected hydrophilic cellulose microfibrils and to intense interaction between the cellulose fibers or nanocrystals. The properties of bacterial cellulose (BC) hydrogels are unique and quite different from those based on plant cellulose because of its ultrafine network structure, high hydrophilicity, and high purity as well as culturing without using any cross linker. Cellulose-based hydrogels can be prepared from wastepaper or various cellulose-containing agricultural wastes. Hydrogels have been extensively studied for a wide variety of applications because their properties and chemical compositions can be easily manipulated. Frequently studied applications of hydrogels are biomedical applications such as drug delivery, wound dressings, and tissue engineering. Additionally, studies have reported evaluating cellulose-based hydrogels as water reservoirs and controlled release fertilizer carriers in horticulture and agriculture [51]. Lately, more technical applications of hydrogels have also been investigated. Cellulose-based hydrogels can be applied as various sensors [52,53,54], heat harvesting [55], fire prevention, and firefighting [56], or in cancer detection or therapy [57,58]. Moreover, cellulose-based composite hydrogels have been studied for the massive uranium extraction from seawater to cope with the severe shortage of onshore uranium reserves [59].

#### 1.2.2. Cellulose Aerogels

Aerogels, on the other hand, are ultra-light weight and highly porous materials that are formed by the replacement of a liquid solvent in a gel by air. In this process, the volume of the gel body or the network structure is maintained, or in other words, the shrinkage phenomena are significantly reduced or eliminated. The interest in these materials originates from their strongly attractive characteristics including low density (typically between 0.004 and 0.500 g cm^−3^), high porosity (typically greater than 80%) as well as high specific surface area, low thermal conductivity, excellent shock absorption, and low dielectric permittivity [50,60].

Cellulose aerogels are prepared from aqueous cellulose suspensions—hydrogels. Subsequent replacement of water with air using freeze-drying (FD) or CO_2_ supercritical drying produces a porous material with the structure displaying entangled cellulose I nanofibers or nanocrystals. In general, the properties of the resulting material depend on several parameters including the initial cellulose nanofiber concentration, drying technique, speed of freezing the suspension before drying, and the chemical or enzymatic pre-treatment of the starting cellulose material. One of the main advantages of using cellulose nanofibers to form the porous material originates from the high reactivity of free hydroxyl groups on their surface. Consequently, the chemical functionalization of cellulosic aerogels represents an attractive approach to tailor the properties of these structures and further broaden their scope of applications. Thus far, two routes have been reported to obtain functionalized porous structures: (1) Preparing the aerogel and modifying it afterward, or (2) derivatizing the nanofibers prior to the formation of the aerogel [13]. Both approaches have been used for the formation of inorganic nanoparticles as well as their organized or assembled structures by applying cellulose as a template. The sources to produce cellulose aerogels are diverse, but cellulose-originated wastes, especially biomass and textile waste, are of primary interest since they are appropriate for the development of eco-friendly production technologies with the use of renewable feedstocks. Aerogels prepared from cellulose-rich waste are biodegradable, easy to functionalize for different uses, and are inexpensive due to the abundance of raw materials [61]. Cellulose aerogels have a wide area of potential applications such as particle filters, particle trappers, catalyst supports, and heat insulators [62,63] and have been evaluated for their capability to remove various pollutants from an aqueous medium [61,64,65]. Additionally, the application of cellulose aerogels for oil absorption applications has also been investigated [66]. Furthermore, cellulose composite aerogels with inorganic nanostructures have also studied been as antibacterial materials for their application in medicine [67,68]. Moreover, a polydopamine-filled cellulose aerogel was developed and studied as a device for seawater and wastewater purification [69]. Another type includes composite aerogels (i.e., cellulose aerogels combined with other materials such as Ag nanowires), which are used to produce materials with a unique combination of mechanical, electrical, magnetic, and thermal properties for application in EMI shielding, electrical switches, and solar–thermal energy conversion [70,71].

The successful design and formation of inorganic nanostructures require effective and convenient methods for their fabrication. In this context, the synthesis of nanomaterials from biopolymers as the templates is a promising approach, which enables the design of complex hierarchical material systems or nanodevices. Celluloses and nanocelluloses are particularly attractive as bio-templating materials because of their highly interactive surface due to a large number of reactive hydroxyl groups that can efficiently nucleate inorganic phases around the rod-like cellulose structures. Cellulose fibers and cellulose nanostructures are supposed to direct the formation, growth, and patterning of inorganic materials to produce various types of nanostructures (nanoparticles, nanowires, or nanotubes) with unique combinations of optical, electrical, and catalytic properties [13]. High open porosity, large surface area, and high mechanical strength make any form of cellulose almost an ideal candidate for the supporting medium in the synthesis of nanostructures.

Recent progress reported in the scientific literature suggests that a synergetic use of the cellulose (nano)fibers together with suitable functionalization pathways will constitute a breakthrough in the formation of templated metallic- and metal oxide hierarchical nanostructures in the near future. Therefore, this review focuses on the utilization of cellulose and nanocellulose materials as templates or building blocks for the formation of nanomaterials and hierarchical functional materials. It provides a review of the applications of any form of cellulose as a template, supporting medium, or growing vector for the formation of inorganic nanostructures. With respect to the presence of cellulose in the final product, these applications can be divided into two groups: cellulose-supported structures and pure inorganic cellulose-templated structures. The first group comprises the publications reporting on the nanostructures where cellulose remains a constitutive component of the final nanocomposite structure, whereas the second group constitutes the publications dealing with the formation of metallic- or metal oxide nanostructures followed by subsequent elimination of the cellulose by calcination, resulting in pure inorganic structures. A special subgroup represents the inorganic nanostructures supported by carbon fibers formed from cellulose fibers by calcination in an inert atmosphere. These two main groups can be further divided based on a type of inorganic material into: metallic nanostructures, metal oxide nanoparticles, metal sulfide and other inorganic compound nanostructures as well as complex hybrid structures containing various metallic and/or metal oxide nanoparticles. For this reason, the review section is divided into five chapters: (2) Cellulose supported metallic nanoparticles; (3) cellulose supported metal oxide nanostructures; (4) cellulose templated pure metal oxide nanostructures; (5) cellulose supported/templated nanostructures of metal sulfides, hydroxyapatites, and other inorganic compounds; and (6) cellulose supported/templated hybrid nanostructures. Each chapter is further organized with respect to the size of the cellulose structures used as a support/template: macrostructured cellulose (cellulose fiber and fibril—CF, regenerated cellulose—RC); microstructured cellulose (microcrystalline cellulose—MCC, microfibrillated or nanofibrillated cellulose—MFC); and nanostructured cellulose (bacterial nanocellulose—BNC, cellulose nanofibril—CNF, cellulose nanocrystal—CNC). The number of publications reporting on cellulose supported or templated inorganic nanostructures in the last 20 years numbers in the hundreds or even thousands. Many research groups have reported on the outstanding capability of any form of cellulose to stabilize and support the growing inorganic nanostructures, thus forming hierarchical nanostructures with exceptional combinations of properties that are practically impossible to prepare in any other way. A large number of publications have suggested that a review is necessary to identify and monitor the trends of where this research area is heading and to list the achievements produced. There have been about ten different review articles published in the last ten years dealing with cellulose and inorganic materials, however, they are limited either by the type of cellulose or by the type of inorganic nanostructures or by the application area of these hybrid nanomaterials. Therefore, this publication offers a review over a wide range of cellulose structures (from cellulose fibers to cellulose nanocrystals) and a wide range of cellulose supported or templated inorganic nanostructures (metals, metal oxides, metal sulfides, hybrid inorganic nanostructures) as well as a wide range of the various application areas of these hierarchical nanomaterials. 

## 2. Cellulose Supported Metallic Nanostructures

Various procedures of the formation of metallic nanostructures on the surface of cellulose have been reported. Authors have frequently used nanocellulose as a metallic nanoparticle support, but other forms of cellulose, even native cellulose fibers, are also useful for this purpose. With respect to the type of metal, mostly noble and particles of some transition metals are deposited on the cellulose surface.

The most abundant metal studied is Ag since it has strong antibacterial activity and together with cellulose structures, it represents almost ideal material for wound dressings or other biomedical applications. There is a substantial number of publications on this topic [72]. Aside from cellulose–Ag, cellulose–Au hybrid materials have also been extensively studied [73]. Cellulose–Au hybrid materials are very attractive due to their chemical inertness and high resistance toward oxidation, making them interesting materials for biomedical applications. Additionally, Ag and Au have been studied as antiviral agents and their hybrid materials with cellulose can be applied in disinfectant wipes or as anti-viral layers in facemasks [74]. This potential application is highly important in the time of the COVID-19 pandemic. Cellulose structures decorated with Pt or Pd nanostructures are lightweight, flexible, environmentally-friendly membranes with strong catalytic activity. Many of the cellulose supported metallic nanostructures have shown strong catalytic activities for numerous chemical reactions [75]. Additionally, these lightweight hierarchical cellulose metallic nanostructures have potential applications in electronics, electro-optics, and chemical sensing. The ether oxygen and the hydroxyl functional groups of the cellulose anchor metal ions tightly on the cellulose fibers via ion–dipole interactions. They also stabilize metallic nanoparticles by the strong bonding interaction with the particle surface, thus enabling the formation of hierarchical metallic nanostructures that are otherwise almost impossible to prepare. Due to the strong interaction between the forming nanoparticles and the cellulose surface, the agglomeration and Ostwald ripening of these nanoparticles are generally prevented. Many authors have reported that cellulose itself possesses reducing properties and the capability to reduce the salts of noble metals to the metallic state, but in most cases, the authors have used reducing agents to form metallic particles on the surface of cellulose. 

**Scheme 1:**
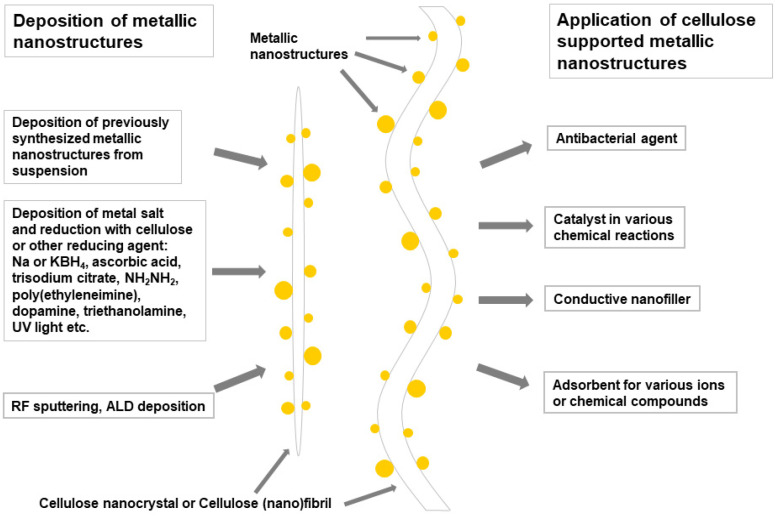
Schematic representation of the cellulose/metallic particle nanocomposites, synthetic routes, and their most frequent applications.

The presentation of the versatility of cellulose substrates, the formation processes of the metallic particles, and the potential application of these hierarchical structures is shown in Scheme 1. 

### 2.1. Cellulose Supported Noble Metallic Nanostructures

Cellulose in any form has been applied as a support or template in the formation of various metallic nanostructures including noble (Ag, Au, Pt, Pd) as well as other transition (Co, Ni, Cu) metals [25]. Theoretically, by using strong reducing agents such as H_2_ or NaBH_4_, any metallic particles, even metallic Fe, can be formed on the cellulose support. On the other hand, noble metals can be reduced to the metallic state by cellulose itself or by using mild biobased reducing agents such as leaf extracts [76].

Various methodologies to synthesize gold and silver nanoparticles on the surface of the cellulose fibers have been explored and compared. Treatment of the cellulose fibers with an alkaline solution of HAuCl_4_ or AgNO_3_ led to the growth and deposition of Au and Ag nanoparticles on their surface. When the cellulose fibers were treated with lecithin or surface modified with thiol, a high temperature was not essential for the growth of nanostructures on the cellulose. With such treatment and modification, uniform metallic nanoparticles were obtained in relatively high yields (~43 wt.% of Au on the cellulose fibers modified with thiol) at room temperature. Reduction with borohydride resulted in substantially lower loading (~22 wt.%) and a wide size distribution of Au and Ag nanostructures on the cellulose fibers. These hierarchical structures were studied as catalysts in the reduction of 4-nitrophenol into 4-aminophenol. Thiol modified cellulose–gold nanoparticle composites quantitatively reduced 4-nitrophenol into 4-aminophenol in 90 min in the presence of NaBH_4_ [77].

Silver nanoparticles (AgNPs) synthesized with four different processes were studied as a disinfectant for cellulose-based wipes. These four techniques are: (1) cotton yarn as a reducing agent with trisodium citrate; (2) cotton fabric as a reducing agent with trisodium citrate; (3) an aqueous solution of PVA in the presence of glucose as a reducing agent; and (4) the photochemical reaction of polyacrylic acid in a AgNO_3_ solution. The prepared AgNPs with particle sizes between 10 and 70 nm were deposited on cellulose fabrics to be used as wipes for the disinfection of surfaces. The assessment of active solutions of silver nanoparticles for the antiviral and antimicrobial activities was evaluated. The results showed a significant effect of the AgNP preparation process on their disinfectant performance. The evaluation of antimicrobial and antiviral activities proved their effectiveness against fungi (*A. niger* and *C. albicans*), coronavirus (MERS-CoV) as well as bacteria (*E. coli* and *P. mirabilis* as Gram-negative bacteria, S. aureus and B. subtilis as Gram-positive bacteria). The AgNPs prepared by using cotton fabric as a reducing agent (2) showed the highest antiviral activity with 51.7% viral inhibition at 0.0625 μL with moderate cytotoxicity activity. Disinfectant cellulose-based wipes treated with antimicrobial and antiviral silver nanoparticles were successfully prepared to be applied for the prevention of the contamination and transmission of several pathogenic viruses (coronavirus) and microbes to humans in critical areas such as hospitals and health care centers [78].

**Figure 1:**
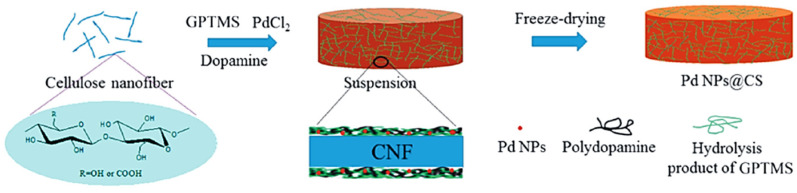
The schematic of the formation of Pd NPs supported by cellulose sponges. Reprinted with permission from [79]. Copyright 2017 American Chemical Society.

Flexible and highly-porous cellulose sponges (CS)-based on the microfibrillated cellulose were prepared by cross-linking with γ-glycidoxypropyltrimethoxysilane (GPTMS) and polydopamine (PDA) and were applied as a support for the synthesis of Pd nanostructures (Pd NPs) (Figure 1). Catechol moieties of polydopamine formed chelates with Pd ions, enabling the nucleation and thus governing the growth of Pd nanostructures on the PDA modified cellulose fibers. The resulting spherical Pd NPs with a narrow particle size distribution between 2 and 6 nm were homogeneously dispersed on the surface of the cellulose fibers. The formed Pd NPs supported by CS exhibited a high catalytic activity in the Suzuki and Heck cross-coupling reactions with almost no leaching of Pd as well as good recyclability. The reported nanostructures represent an innovative approach toward the formation of highly effective, non-leaching, and recyclable metallic heterogeneous catalytic systems [79].

Microcrystalline cellulose was applied as a cellulose support in the microwave-assisted synthesis of Ag nanostructures from the AgNO_3_ precursor using ascorbic acid (AAc) as a reducing agent to prepare cellulose–Ag nanocomposites. The properties of the nanocomposites were studied as a function of the microwave heating time and AAc concentration, and the average particle site of the Ag nanostructures varied between 50 and 250 nm. The results confirmed that the AAc concentration played an important role in the nanostructure formation, and a homogeneously decorated cellulose surface by Ag nanostructures was obtained. Since such a microwave-assisted method can be carried out without any seed or surfactant, it represents a fast and convenient approach to a cost-effective and large-scale production of cellulose–Ag nanocomposites [80].

A novel method was developed to synthesize and deposit silver nano particles (AgNPs) on the surface of bacterial cellulose (BNC) by applying polyvinyl alcohol (PVA) to form a nano porous matrix and to reduce the cytotoxicity of the deposited silver nanoparticles in comparison with those synthesized by the chemical reduction. Polyvinyl alcohol was used to reduce the absorbed silver ions (Ag^+^) on the surface of the BNC to the metallic silver nanoparticles (Ag^0^). The size and size distribution were controlled by adjusting the molar ratio of PVA:AgNO_3_. At the optimized reaction conditions, well dispersed and regular spherical AgNPS were formed with particle sizes ranging from 15 to 35 nm. The AgNPs displayed an optical absorption band around 420 nm. The cytotoxicity of these AgNPs were determined by using the MTT assay and the IC_50_ values to demonstrate the effect of the AgNP preparation method on the cytotoxicity of the formed AgNPs on the A549 viable cells. The results showed that PVA and BNC significantly reduced the concentration of Ag^+^ ions on the contact surface with A549 cells, thus significantly reducing the cytotoxicity of the so-prepared hybrid AgNPs–PVA–BNC materials [81].

An innovative cellulose–palladium nanocatalytical system (cell-OOCPhPPh_2_-Pd) has been developed using a convenient synthesis approach and simple precursors. Microcrystalline cellulose (MCC) was first tosylated and further reacted with 2-(diphenylphosphino) benzoic acid to produce microcrystalline cellulose–phosphinite. This modified MCC was then reacted with Pd(OCH_3_)_2_ to produce Pd doped MCC–phosphinite. Pd nanostructures have sizes from 1 to 5 nm and showed high activity in C–H activation as well as in three other types of cross-coupling reactions (Suzuki–Miyaura, Heck, and Sonogashira). All types of transformations were carried out with a single type of nanocatalyst and the yields were higher than 60%. The reaction products contained low Pd residues and the studied catalyst was readily recycled by simple filtration without significant loss in the catalytic activity, even after several runs. The above features suggest that this catalyst is highly promising for applications in the pharmaceutical industry [82].

Innovative composites of bacterial cellulose and Au nanoparticles were formed by a single-step biotemplated process in an aqueous suspension by applying polyethyleneimine (PEI) as a reducing agent. The thickness of the Au shell in the Au–BC nanocomposites was controlled by adding various halides, while the PEI, adsorbed to the surface of the BC nanofibers via hydrogen bonding, functioned both as a reducing agent and as a linker for the Au nanostructures. The average particle size of the grown Au nanoparticles was between 5 and 20 nm. Subsequently, horseradish peroxidase (HRP) was embedded into the fibrous structure of the Au–BC nanocomposites without any reduction in its bioactivity. Thus, the prepared HRP biosensor demonstrated a highly-sensitive detection of H_2_O_2_ with a detection limit close to 1 μM. The obtained Au–BC nanocomposites offer a promising support for the immobilization of other enzymes and enable biosensor fabrication for a wide range of applications in bioelectrocatalysis and bioelectroanalysis [83].

**Figure 2:**
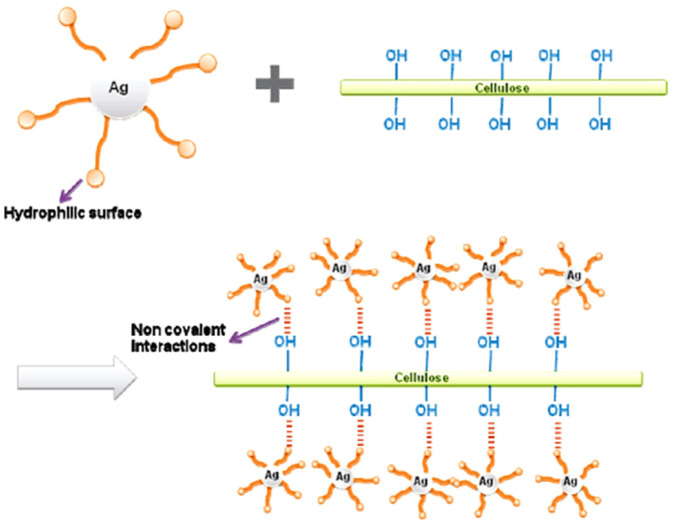
The synthesis mechanism for the formation of Ag nanoparticles on the surface of the CNCs. Reprinted with permission from [16]. Copyright 2010 American Chemical Society.

On many occasions, the authors used surfactants as stabilizers or agents for positioning particles on the cellulose surface. For example, tunicate CNCs were modified on their surface with metallic nanoparticles using the cationic surfactant cetyltrimethylammonium chloride (CTAB), which served as a stabilizer of the metallic nanoparticles and as a distributing agent for the nanoparticles on the surface of the CNCs (Figure 2). This synthetic path enabled the effective formation of Pt, Au, Cu, and Ag nanoparticles on the surface of the CNCs. With respect to the particle size distribution, the nanoparticles were polydisperse, which was ascribed to the competition of two processes, nucleation and particle growth, which governed their formation. The nanoparticles’ average size (5–20 nm) and the thickness of the metallic layer on the CNCs were both controlled by the pH of the salt solution, the reduction time, and the concentration of CTAB. The applied CTAB stabilized the metallic nanoparticles and increased their interaction with the cellulosic substrate, resulting in the enhanced coverage of the surface of the CNCs with metallic nanostructures [16].

**Figure 3:**
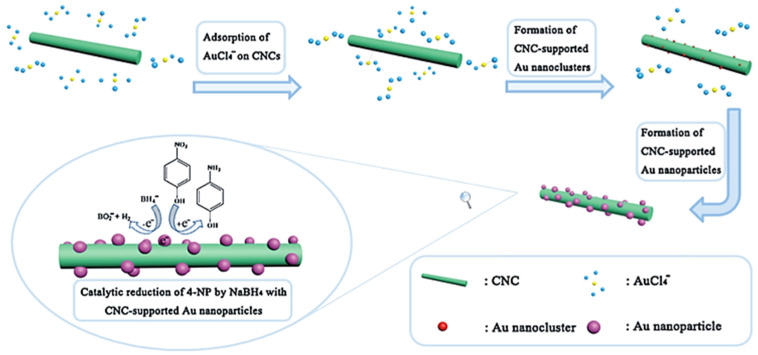
The schematic illustration of the formation process of CNC-supported Au NPs and their catalytic activity in the reduction of nitrophenol. Reprinted from [84]. Copyright 2015, with permission from Elsevier Ltd.

CNCs were used as a support to synthesize gold nanoparticles (Au NPs) by heating the aqueous mixture of polyethylene glycol, CNCs, and HAuCl_4_ at 80 °C for 1 h. In this way, extreme conditions and toxic chemicals as well as a complicated procedure were avoided (Figure 3). The prepared CNC-supported Au NPs demonstrated high catalytic activity for the reduction of 4-nitrophenol using sodium borohydride. The maximum turnover frequency and the apparent rate constant reached 641 h^−1^ and 1.47 × 10^−2^ s^−1^, respectively. Due to the large concentration of highly dispersed Au NPs (average size 2–15 nm) that was deposited onto the CNC surface, a high catalytic performance was observed. The reported procedure proved to be environmentally-friendly, low-cost, and simple and has a large potential in medical and industrial applications [84]. 

### 2.2. Cellulose Supported Non-Precious Metallic Nanostructures

Aside from noble metal nanoparticles, semi- and non-precious metallic nanoparticles have also been successfully formed on the surface of various cellulose structures. A green synthetic approach to prepare cobalt/cellulose nanocomposites with antibacterial and magnetic properties has been developed by the in situ reduction of cobalt nitrate on the substrate surface using hydrogen gas or NaBH_4_ as reducing agents. Spherical, cellulose-stabilized cobalt metallic nanoclusters with an average diameter of 7 nm were formed with cubic cobalt (α-cobalt) as the main component when hydrogen gas was used as a reducing agent. The cellulose-stabilized, metallic cobalt nanoclusters were homogenously distributed on the surface of the cellulose fibers. The cobalt/cellulose nanocomposites were contaminated with almost insignificant traces of boron. In contrast, the in situ reduction of cobalt ions on the cellulose surface with sodium borohydride leads to amorphous cobalt/cellulose composites with a significant contamination of boron. All of the cobalt/cellulose nanocomposites showed significant antibacterial action against the tested bacterial isolates [85].

CNCs were used as a support in the growth of copper nanoparticles (Cu NPs) by the reduction of CuSO_4_·5H_2_O, representing an environmentally-friendly, low-cost, and simple preparation method. Aqueous NaOH, ascorbic acid, and hydrazine were used as the pH controller, antioxidant, and reducing agent, respectively. The product was high purity spherical Cu NPs with the average size of ~3 nm. Compared to the unsupported Cu NPs, the reported Cu NPs–NCC demonstrated superior catalytic activity and high sustainability for the reduction of methylene blue in an aqueous solution of NaBH_4_ at RT. The prepared Cu NPs–NCC quantitatively reduced the methylene blue in the reaction time of 12 min with the rate constant of 0.7421 min^−1^ and the correlation coefficient (*R2*) of 0.9922 [86].

CNCs were applied as a support in the formation of metallic iron nanoparticles (Fe NPs, 3–10 nm or 15–40 nm, depending on the quantity of CNCs) by an environmentally-friendly single-step procedure. Hydroxyl groups on the surface of the CNCs served as anchor points for the stabilization of Fe NPs and as a reducing agent in the growth of Fe NPs. Additionally, CNCs acted as corrosion inhibitors for metallic Fe NPs and increased their catalytic activity since they retained the metallic state even after 5 days of air exposure. The CNC-stabilized Fe NPs exhibited a catalytic activity toward the methylene blue degradation. It was demonstrated to be a promising and nontoxic nanocatalyst for the hydrogenation reaction of 4-nitrophenol into 4-aminophenol. Additionally, the autonomous motion of the CNC-supported Fe NPs was observed in the presence of H_2_O_2_ and their trajectories were controlled externally by the pH gradient and magnetic field. It was demonstrated that their speed and controlled trajectory could be remotely tuned, making these nanomotors potential innovative nanomachines in drug delivery, imaging applications, and as sensors [87].

The number of publications on metallic nanostructures supported and templated by cellulose structures is vast and not all of them can be included within this review. Therefore, the additional information on publications dealing with the cellulose-metallic nanostructures is summarized in Table 1. Most commonly, the cellulose is decorated or coated with noble metal (Au and Ag) nanostructures and the most widely used reducing agent is NaBH_4_, or simply cellulose itself, while frequent applications of these hierarchical structures are in catalysis, medicine (as antibacterial agents), and electronics (as conductors) (Table 1). The most common method of deposition is the adsorption of metallic ions to the cellulose surface and their subsequent reduction to metallic particles. Alternative approaches include the separate formation of metallic nanoparticles and their subsequent deposition to cellulose fibers, or physical methods such as atomic layer deposition or plasma sputtering.

## 3. Cellulose Supported Metal Oxide Nanostructures

Cellulose structures were used as supports and templates for the formation of various metal oxide nanostructures. In contrast to metallic nanostructures that are mainly limited to noble or semi-noble metals, practically any type of metal oxide can be deposited on the cellulose surface with TiO_2_, Fe_3_O_4_, and ZnO being the most abundant. For this purpose, various cellulose types have been applied, spanning from natural cellulose fibers to cellulose nanocrystals in the original or chemically modified form.

**Scheme 2:**
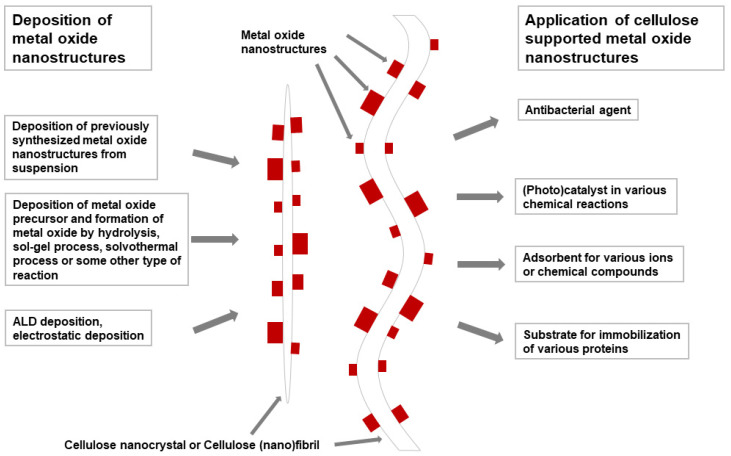
The schematic representation of the cellulose/metal oxide particle nanocomposites, the synthetic routes, and the most frequent applications of such materials.

Various metal oxide nanostructures were deposited or formed on the cellulose surface that serves as the support and template in the formation of nanostructures. By using cellulose templates, various hierarchically organized nanostructured metal oxide particles were synthesized. The most widely used metallic oxides have been TiO_2_, ZnO, Fe_3_O_4_, and Fe_2_O_3_. Using cellulose as a support enables the formation of lightweight, highly flexible, porous, mechanically resistant, and environmentally-friendly structures accompanied with interesting catalytic, electronic, optical, magnetic, and antibacterial properties. The principal application of such structures have been found in heterogeneous catalysis and in the removal of toxic heavy metal ions (Pb^2+^, As^5+^, Mn^2+^, and Cr^3+^) from water. In particular, for the latter application, cellulose structures are particularly suitable since a comparison of the heavy metal ion absorption capacities of various inorganic sorbents and organically-modified cellulose structures is significantly in favor of the latter ones [153]. Cellulose gels with their specific structure, high specific surface, and porosity as well as a high concentration of functional groups are very suitable for the removal of various pollutants from water [153]. The incorporation of various inorganic compounds into cellulose gels enhances the pollutant removal capability and efficiency, and introduces a specific affinity toward certain pollutants. Aquatic pollutants that can be removed in this way comprise various metal ions, organic dyes, and some anions. Additionally, the ZnO/cellulose hierarchical nanostructures show a high applicative potential in medicine as wound protective materials due to the antibacterial properties of nano ZnO. Moreover, CuO, Cu_2_O, ZnO, and TiO_2_ were studied as antiviral agents and their hybrid materials with cellulose are potentially applicable in disinfectant wipes or as anti-viral layers in facemasks [74]. For example, CuO showed an intense antiviral activity and such hybrid nanomaterials were applied as an antiviral protection layer in respiratory facemasks. The practical tests demonstrated that CuO impregnated masks safely reduced the risk of influenza virus environmental contamination without altering the filtration capacities of the masks and that the manufacture of the mask with CuO layers would not add any significant costs to the price of such masks [154]. These applications are highly important nowadays, leading to the development of masks with improved protection against COVID-19. On the other hand, Fe_3_O_4_/cellulose structures are lightweight, flexible, magnetic materials that offer a wide range of potential applications. Cellulose can stabilize not only the metallic nanoparticles, but also the metal oxide nanoparticles by the strong bonding interaction of metal oxide nanoparticles with the cellulose surface, enabling the formation of hierarchical metallic oxide nanostructures that are otherwise practically impossible to prepare. The presentation of the versatility of cellulose substrates, the formation processes of metal oxide nanostructures, and the potential applications of these hierarchical structures is given in Scheme 2. 

### 3.1. Cellulose Supported TiO_2_

The most frequently used metallic oxide for the decoration of the cellulose surface is TiO_2_, since it offers various functionalities (photocatalytic activity, energy conversion, heavy metal absorption, etc.) and a broad range of potential applications including self-cleaning and dynamically responsive materials. Rutile TiO_2_ nanoparticles (average particle size 100–250 nm) have been prepared via in situ synthesis using cellulose fibers as a support to form cellulose fiber/nano-TiO_2_ composites. Additionally, CF/TiO_2_ was also prepared by the electrostatic deposition of commercial TiO_2_. Subsequently, composite membrane beds were prepared using a wet-laid procedure. After this, the dynamic Pb^2+^ adsorption was studied by passing the feed solution through the prepared beds using a single-pass flow mode. The impacts of parameters such as the bed stacking pattern, flow rate, and bed height on the bed breakthrough performance were studied. The results demonstrated that the CF/in situ-TiO_2_ bed had more favorable characteristics than the others, since its Pb^2+^ loading capacity was 9-times higher than that of the CF/self-assembled TiO_2_ bed and 13-times higher than that of the pure CF bed before 10% breakthrough occurred. Moreover, this bed showed high selectivity for Pb^2+^ toward Ca^2+^, and was easily regenerated for repeated use by rinsing with 0.1 M HCl solution. The excellent performance of the in situ-TiO_2_/CF bed was ascribed to its faster adsorption kinetics and larger fiber adsorption capacity compared to other types of beds with equal bed structures [155].

**Figure 4:**
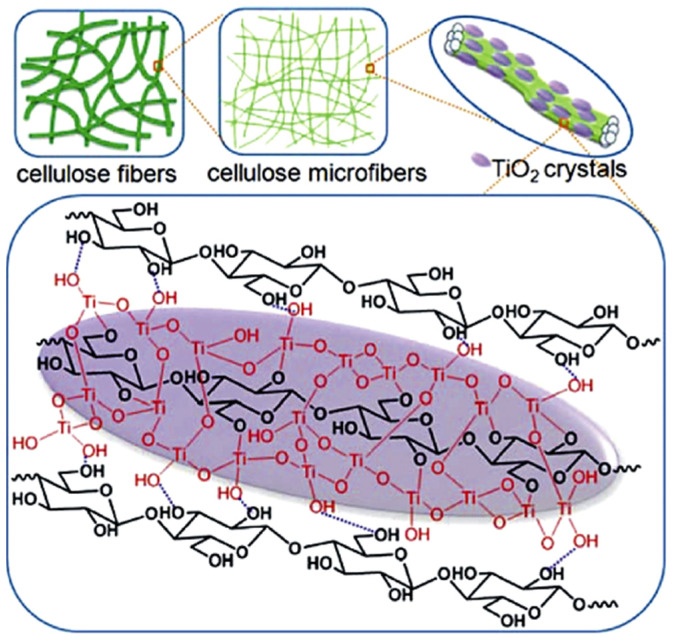
The schematic illustration of the in situ directional growth of spindle TiO_2_ along the cellulose microfibers. Reprinted from [156]. Copyright 2015, with permission from Elsevier Ltd.

TiO_2_ nanocrystals (TiO_2_ NCs) were formed in situ on the surface of the cellulose fibers (CFs) by a simple hydrolysis of TiOSO_4_ to prepare the cellulose/TiO_2_ nanocomposites. Initially, cellulose was applied as a scaffold for the TiO_2_ NC immobilization. However, the results showed that CF also functioned as a chemical template, directing the crystal growth of TiO_2_ (Figure 4). Consequently, uniformly dispersed spindle rutile TiO_2_ crystals (length ~180 nm and width ~50 nm) were formed on the CF surface, which exhibited a tendency to the adsorption of heavy metal ions such as Pb^2+^. The CF/TiO_2_ composites demonstrated an improved high selectivity for lead (Pb^2+^) removal, high adsorption capacity, and good regenerability. Nonwoven filter beds were easily prepared using the prepared composite fibers that were further used in the dynamic filtration experiment. The CF/TiO_2_ beds showed a 12-fold increase in the filtered bed volume before the breakthrough occurred when compared to that of the pure CF beds. This study represents a green route toward the preparation of highly efficient and low-cost nanosorbents for water decontamination using CF as the support and template [156].

The TiO_2_ nanostructures (particle size 2–10 nm) were formed by in situ precipitation in the micronanoporous structure of the regenerated cellulose matrix using a sol–gel method to prepare the titania/cellulose composite films. The titania nanoparticles were formed inside the cellulose matrix, which provided reaction nanocavities for the formation of TiO_2_ nanostructures and hydroxyl groups for their interactive adsorption. The composite films showed high photocatalytic activity in the degradation reaction of phenol using low intensity UV light. The nano TiO_2_/regenerated cellulose films with interesting mechanical properties proved to be an efficient and reusable catalyst in the photodegradation of organic pollutants such as phenol [157].

A thin TiO_2_ film was formed on lightweight native nanocellulose aerogels using a chemical vapor deposition method (CVD), producing a new type of photo switching functional material between water-repelling and water-absorbing states. Uniform titania coatings with an average thickness of ~7 nm were prepared on cellulose fibers of the aerogel. As shown by weighing, the TiO_2_-coated aerogel samples practically did not absorb water during exposure, which was also proven by a high surface contact angle for water of 140°. After UV exposure, they intensively absorbed water (16-times their own weight) and showed a very low surface contact angle that is characteristic of superabsorbents. The recovery of the original properties was observed after storage in the dark for 2 weeks. Additionally, the TiO_2_-coated nanocellulose aerogels demonstrated a photocatalytic activity in the photooxidative decomposition of methylene blue, which, combined with the highly porous structure, provides a high potential in water purification applications [158].

**Figure 5:**
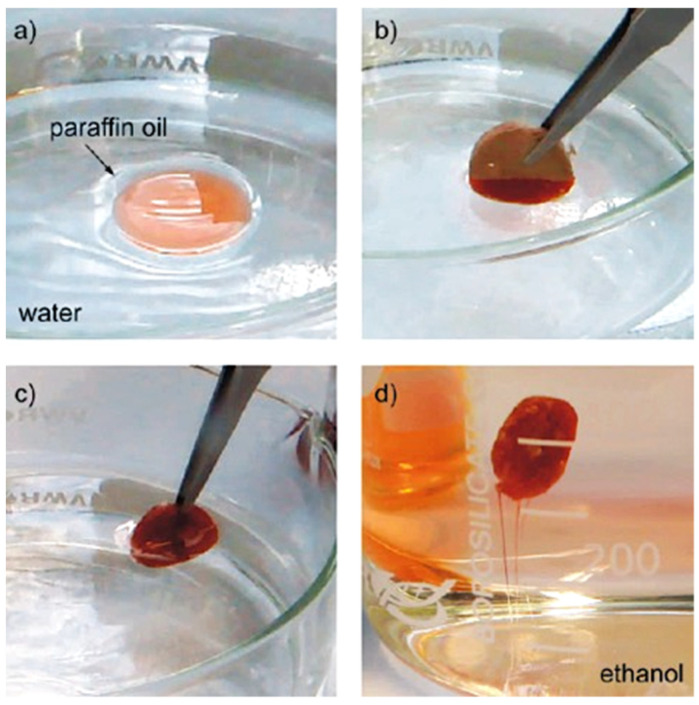
Oil spill removal from water. Paraffin oil (colored for clarity) floating on water (**a**); oil being absorbed into the aerogel (**b**); all of the floating oil has been absorbed (**c**); oil-filled aerogel can be washed simply by immersing it in a solvent such as ethanol. The oil is removed as shown by the red streaks (**d**). Reprinted with permission from [159]. Copyright 2010 American Chemical Society.

Cellulosic aerogels have been modified with TiO_2_ nanoparticles using a chemical vapor deposition process, introducing oleophobic functionality in such systems. It was demonstrated that functionalization of the native cellulose nanofibrils of the aerogel with an oleophilic and hydrophobic titania coating produced a water floating and selectively oil-absorbing material. These surface modified aerogels enabled the removal of organic contaminants from the water surface due to the low density combined with the capability to absorb large amounts of nonpolar liquids and oils (Figure 5), and moreover, they were recyclable and reusable after washing or alternatively, they could be incinerated together with the absorbed liquid [159].

### 3.2. Cellulose Supported Fe_3_O_4_ and Other Iron Oxides

Cellulose structures are often decorated with Fe_3_O_4_ to introduce magnetic properties on the cellulose surface for various applications including magnetic resonance imaging for enzyme immobilization, clinical diagnosis, hyperthermia anticancer treatment, and magnetic drug targeting. Iron oxides such as magnetite (Fe_3_O_4_) and maghemite (γ-Fe_2_O_3_) have been proposed for these technological applications because of their high magnetic transition temperatures, high saturation magnetization, and their biocompatibility. In particular, water-dispersible polymer-based magnetic micrometer-sized spheres have attracted high attention for immobilizing various biopolymers such as enzymes and other drugs for diagnostic applications, controlled drug delivery, cell labeling, and separation purposes. 

Fe_3_O_4_ nanoparticles (particle size 5–12 nm) were precipitated on the surface of the regenerated cellulose fibers to prepare nano-magnetic cellulose (MCGT) with excellent magnetic properties during the process of cellulose regeneration. Subsequently, a highly efficient adsorbent was formed by grafting MCGT with glycidyl methacrylate and tetraethylenepentamine and the final product was evaluated for the removal of Ag(I), Cu(II), and Hg(II). The measured adsorption capacities were 1.2, 1.5, and 2 mmol g^−1^ for the Ag(I), Cu(II), and Hg(II), respectively. The kinetic studies revealed that adsorption followed the pseudo-second-order kinetic model, whereby the adsorption equilibrium was reached within 3–5 min. The thermodynamic parameters revealed the spontaneous and exothermic adsorption of ions. Grafted MCGT was easily separated and regenerated from the adsorption medium by using an external magnetic field while thiourea was applied for the removal of adsorbents. It was tested for a real industrial wastewater treatment, demonstrating a high removal efficiency of pollutants, making the prepared MCGT a promising substrate for these purposes [160].

Fe_3_O_4_ spherical nanoparticles were formed on the bacterial cellulose (BC) and new BC/Fe_3_O_4_ nanocomposites (BC/Fe_3_O_4_) were prepared during BC biosynthesis from the *G. xylinum* fermentation by using an innovative pH controlling method. The fermentation produced Fe_3_O_4_ nanoparticles with the average diameter of 15 nm being uniformly distributed in BC. The BC/Fe_3_O_4_ nanocomposites with a 33 wt.% of Fe content showed the saturated magnetization (*σ_s_*) of 41 emu g^−1^ and the coercivity of 27 Oe. The adsorption and elution characteristics of the BC/Fe_3_O_4_ nanocomposites for the Cr^3+^, Pb^2+^, and Mn^2+^ ions were studied. Their adsorption capacities were found to differ significantly at the same concentration of an ion and they were ranked in the order Cr^3+^ < Mn^2+^ < Pb^2+^, whereas the sequence of the elution capacity followed the order Cr^3+^ < Pb^2+^ < Mn^2+^. The BC/Fe_3_O_4_ nanocomposites were recyclable by applying magnetic field separation after the elution of the adsorbed heavy metal ions [161].

Never dried bacterial cellulose (BC) nanofibrils were surface functionalized with aminated magnetite nanoparticles (AMH). Particles were formed by the solvothermal reaction of FeCl_3_, 1,6-hexanediamine, and BC pellicles in ethylene glycol medium at 200 °C with a 6 h reaction time. Cellulose accelerated the growth of Fe_3_O_4_ nanostructures and facilitated their deposition on the cellulose nanofibers. The AMH attached on the cellulose nanofibril surface significantly improved the mechanical and thermal properties as well as increased the amine concentration of the BC pellicles. The BC–AMH pellicles exhibited a high adsorption capacity toward the As^5+^ ions due to their high AMH contents. Almost 90 mg of As^5+^ per gram of the BC–AMH composite was achieved. The high mechanical and chemical stability, large surface area as well as the high content of surface amine groups in the BC–AMH pellicles show that these structures have potential in applications such as enzyme immobilization, drug delivery, and catalysis [162].

**Figure 6:**
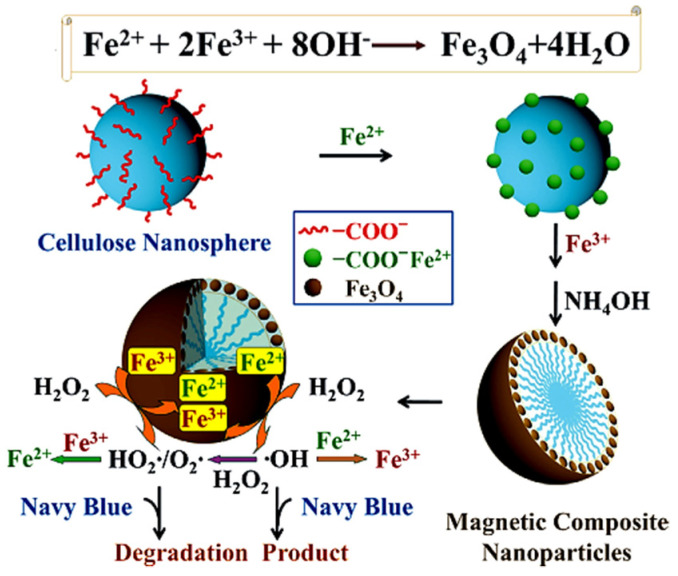
The formation and catalytic mechanism of MNPs. Magnetic iron oxides were anchored on the cellulose nanospheres by using a co-precipitation process; the rapid removal of textile dye Navy blue was achieved in the Fenton-like system. Reprinted from [163]. Copyright 2015, with permission from Elsevier Ltd.

The Fe_3_O_4_ nanostructures were deposited on the surface of the carboxyl cellulosic nanospheres by using a simple co-precipitation process to form magnetic cellulose nanocrystal particles (MNPs) (Figure 6). The morphology and structure of the Fe_3_O_4_ nanoparticles (size ~50 nm) were controlled by cellulosic nanospheres that functioned as a template for their stabilization. The MNPs were efficiently recycled and reused by using an external magnetic field. The spherical MNPs showed a superparamagnetic behavior accompanied by a sensitive magnetic response under an external magnetic field, and functioned as highly efficient nanocatalysts in the Fenton-like system for the rapid removal of Navy blue textile dye from an aqueous solution. By using MNPs that were prepared with H_2_O_2_ at the feed ratio of 1:2, 90.6% of Navy blue was removed in the first min and 98.0% in 5 min time, while 75.0% of the removal efficiency was preserved in the fifth cycle by optimizing the reaction conditions. Because of the low-cost, large surface area, good dispersibility, strong catalytic activity, and facile preparation as well as the ability of magnetic recycling and reuse, the reported MNPs showed potential application as highly effective heterogeneous nanocatalysts [163].

### 3.3. Cellulose Supported ZnO

A frequently used metal oxide for cellulose decoration is definitely ZnO due to its wide range of outstanding physical and chemical properties. Optical and electrical properties make it attractive in various optical, electrical, and electro-optical applications, whereas chemical properties make it highly promising in a large number of catalytical and accelerating applications. Finally, the antibacterial properties of ZnO show that it has high potential in various medical and other protective applications.

The ZnO nanostructures were formed on regenerated cotton cellulose by a simple one-pot wet chemical method using the Zn–amine complex to prepare nano ZnO–cellulose hybrid materials. The Zn–amine complex was prepared from ZnNO_3_ as a precursor and triethanolamine without thermal treatment. The hybrid material contained around 40 wt.% of ZnO nanoparticles (size ~250 nm) with a uniform distribution of nano ZnO through the cross section of the material. The ZnO–cellulose hybrid film was formed by the initial adsorption of Zn^2+^ ions to the surface of the hydrophilic cellulose fibrils, followed by the alkaline hydrolysis of the Zn–amine complex and the growth of ZnO nanostructures. Possible applications of the reported nano ZnO–cellulose hybrids are in optoelectronics as flexible display devices and biomedical or strain sensors [164].

The ZnO nanoparticles were formed simultaneously with the ZnO/cellulose nanocrystal (ZnO/CNC) nanohybrids by the hydrolysis of commercial microcrystalline cellulose (MCC) with citric/hydrochloric acid, followed by precipitation using an aqueous solution of zinc nitrate. The prepared carboxylated CNCs functioned as the stabilizing and supporting agent for the growing ZnO nanoparticles, anchoring them to the surface. ZnO nanostructures with hexagonal wurtzite structure with an average diameter of 42.6 nm were deposited onto the surface of the CNCs. The prepared ZnO/CNC nanohybrids demonstrated a high antibacterial activity and improved the thermal stability and photocatalytic activity for methylene blue (MB) as 93% of MB was decomposed after the UV light exposure within 100 min [165]. Therefore, the reported ZnO/CNC hybrids are promising photocatalytic or biomedical materials.

Spherical nanocrystalline ZnO particles with an average size of 100 nm have been formed through a simple in situ polyol method by using an amidoxime modified bacterial cellulose (Am–BC) as a template. The hydroxyl and amidoxime groups of the Am–BC functioned as reactive sites for the growth of zinc oxide NPs. The Am–BC nanoreactor as a template produced ZnO NPs in a much higher yield compared to those based on the unmodified BC. Moreover, Am–BC prevented the aggregation of ZnO NPs, resulting in well-dispersed and regular ZnO NPs assembled in situ into the Am–BC network, which showed excellent photocatalytic properties. The reported synthetic process represents a simple and effective approach for the formation of other metallic nanostructures [166].

**Figure 7:**
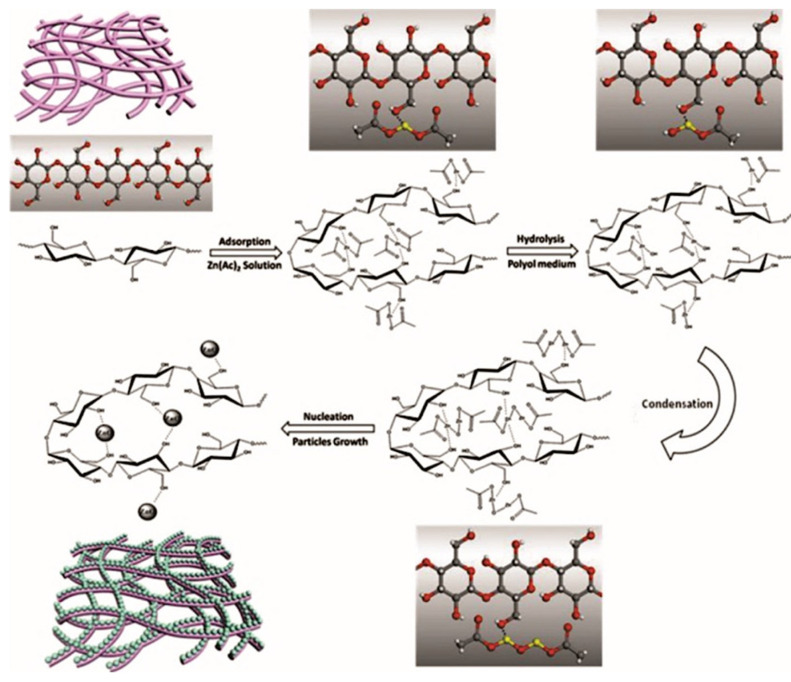
The schematic illustration of the formation of the ZnO nanoparticles impregnated into the BC [167]. Copyright 2013, with permission from Elsevier Ltd.

The BC was applied as a bio-template in the precipitation of crystalline ZnO nanoparticles by a simple polyol method (Figure 7). Their particle size and morphology were to a large extent affected by the hydrolysis time and concentration of the Zn(OOCCH_3_)_2_ precursor. Under the optimal reaction conditions (hydrolysis time: 10 min, concentration of Zn^2+^:0.5 wt.%), nano ZnO with an average size of 100 nm was formed. The BET surface areas of the pure BC fibers and the ZnO/BC composites were found to be 55.36 and 101.34 m^2^ g^−1^, respectively, indicating these structures as promising photocatalytic materials. The obtained ZnO/BC composites showed a relatively high photocatalytic activity with a maximal decolorization efficiency of 70% at 0.5 wt.% of Zn^2+^ [167].

By using an ultrasonic-assisted in situ synthesis, nano ZnO particles were formed and incorporated simultaneously into bacterial cellulose (BC) pellicles. The BC pellicles were initially dispersed in a zinc acetate solution, followed by the addition of Zn^2+^-absorbed BC pellicles to the ammonium hydroxide solution accompanied by simultaneous ultrasonic treatment. The influences of the immersion time of the BC pellicles in the zinc acetate solution as well as the ultrasonic exposure time on the weight percent and particle size of the deposited nano ZnO were studied. The average particle size of the ZnO formed within the BC pellicles was in the range of 54–63 nm. The ZnO particles deposited inside the BC showed an unusual morphology, which was explained by the growth, with their direction being perpendicular to the alignment of the BC nanofibrils. The prepared BC-nano ZnO sheets showed a high antibacterial activity against Gram-negative and Gram-positive bacteria. The reported BC sheets decorated with nano ZnO are promising materials for effective antibacterial wound dressings [168].

Composites of regenerated bacterial cellulose (RBC) with nano ZnO (ZnO-RBC) were prepared for enhanced biomedical applications by using previously synthesized ZnO NPs. Various concentrations of ZnO NPs were admixed to the solution of RBC dissolved in N-methylmorpholine-N-oxide, and RBC nanocomposite films containing 1 and 2 wt.% of ZnO were cast from the solutions by using an applicator. The prepared ZnO–RBC nanocomposites showed substantially improved mechanical, thermal, and biological properties. Moreover, the testing of the ZnO–RBC nanocomposites revealed significantly enhanced antibacterial properties and materials that were biocompatible with excellent cell adhesion capabilities. The potential applications of the studied ZnO–RBC nanocomposites are in bioelectroanalysis and other biomedical fields [169]. 

Flower-like zinc oxide (ZnO) nanorod clusters were grown on the surface of spherical cellulose nanocrystals (SCNs with cellulose II crystal structure) applied as a growth substrate by a simple chemical precipitation process. In strongly alkaline (pH = 11) conditions, flower-like ZnO nanorod cluster nanohybrids were grown at a temperature of 100 °C in 2 h of reaction time. On the other hand, rod-like ZnO particles were formed at a temperature of 90 °C in weakly alkaline (pH = 9.3 and 10.5) conditions. A possible growth mechanism for the flower-like ZnO on SCNs is that by increasing the NH_3_ concentration (pH = 11), a larger number of hydroxyl groups favors the formation of Zn(OH)_4_^2−^ rather than Zn(NH_3_)_4_^2+^. This restricts the continuous growth of ZnO nanorods, resulting in the formation of new ZnO nuclei on sites of the SCN surface, and finally, in the growth of flower-like ZnO nanorod clusters. The flower-like nanohybrids exhibited enhanced antibacterial activity against *E. coli* and *S. aureus*, and higher photocatalytic activity compared to the rod-like nanohybrids and commercial ZnO [170].

### 3.4. Cellulose Supported Miscellaneous Inorganic Oxides

Additionally, other metallic oxides have been successfully formed on the cellulose surface for various purposes. For example, Cu_2_O was formed by an innovative synthetic approach that exploited the dual properties of the thiol-grafted cellulose paper and was developed for promoting copper-catalyzed [3+2]-cycloadditions of organic azides with alkynes (Click reaction) and for adsorbing/eliminating of the residual copper species from the reaction solution. The cellulose paper was functionalized with thiol (-SH) groups by grafting the cellulose with thioglycolic acid. The -SH grafted cellulose (Cel–SH) had a dual function, that is, promoting the Cu-catalyzed Huisgen synthesis of 1,4-disubstituted triazoles through the reduction of CuSO_4_·5H_2_O to Cu_2_O as well as adsorbing and eliminating copper byproducts from the reaction medium. The thiol-modified cellulose paper reduced Cu^II^ to catalytically active Cu^I^ and acted as an effective adsorbent for copper, thereby simplifying the synthetic process and resulting in the reaction mixture being almost free of copper residues after a single filtration (Table 2). Highly complex organic molecules were synthesized to demonstrate the robustness of the reported cellulose-supported catalytic system as well as the related synthetic approach [171].

Magnetic CoFe_2_O_4_ nanoparticles were deposited into the cellulose structure to prepare lightweight and flexible magnetic cellulose aerogels by a simple method. Their porosities varied from 52 to 78%, while their density and internal specific surface area were between 0.25–0.39 g cm^−3^ and 300–320 m^2^ g^−1^, respectively. The concentration of the incorporated CoFe_2_O_4_ nanoparticles increased by increasing the precursor concentration, however, the particle size practically did not change. The deposited CoFe_2_O_4_ nanoparticles showed a superparamagnetic behavior as well as interesting mechanical properties and changed the microstructure of the cellulose aerogels. Because of the cellulose sustainability and availability in large quantities, the reported process with a simple concept is suitable for the industrial-scale up and is also applicable for many other types of nanoparticles [172].

The lightweight porous magnetic aerogels were prepared from the bacterial cellulose (BC) aerogels as templates, which were compacted into a stiff magnetic nanopaper. Non-agglomerated ferromagnetic CoFe_2_O_4_ nanostructures, with diameters ranging from 40 to 120 nm, were formed from FeSO_4_ and CoCl_2_ precursors in a mixture of NaOH and KNO_3_ on BC nanofibrils functioning as templates. The reported dry magnetic aerogels were flexible, porous (98%), lightweight, and they were actuated by a small magnet to absorb water and release it during the compression. Due to their high porosity, large surface area, and flexibility, these aerogels are promising materials for electronic actuators and microfluidic device applications [173].

**Figure 8:**
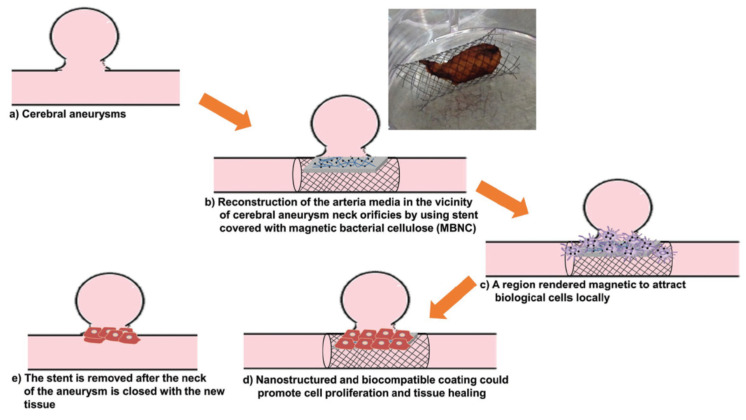
The schematic presentation of the concept of endovascular treatment at the neck orifice in cerebral aneurisms (**a**). The process consists of arterial media reconstruction by using a stent covered with the magnetic bacterial nanocellulose (MBNC) (**b**) designed to attract cells to the aneurysm region (**c**). With time, new tissue grows over the stent, closing the neck of the aneurysm (**d**) and ultimately, the removal of the stent (**e**). The photographic image of the Nitinol stent covered with MBNC revealed their good adhesion to the stent [174]. Copyright 2017 John Wiley & Sons Inc. Reproduced with permission.

An alternative method for the treatment of brain aneurysms was developed by applying magnetized bacterial nanocellulose (BNC) as a platform for both magnetic and cell attraction as well as for cell proliferation (Figure 8). The preparation of magnetic bacterial nanocellulose (MBNC) was carried out via the reaction of Fe II and Fe III precursors, precipitating superparamagnetic iron oxide nanoparticles, which were afterward coated with polyethylene glycol to enhance their biocompatibility. The biocompatibility and cytotoxicity testing of MBNC showed suitable cell viability and minimal cytotoxicity. The study of cellular magnetic attraction and the cell migration tests revealed the viability of MBNC as a multifunctional coating of stents for the treatment of brain aneurism [174]. 

Cellulose nanocrystal (CNC) films and electrospun composite CNC fibers with magnetic properties were prepared. Magnetism was introduced into the CNC by CoFe_2_O_4_ nanoparticles immobilized on the CNC nanofibrils. The CNCs (ca. 150 nm length) and the aqueous dispersion of the cobalt–iron oxide nanoparticles (10–80 nm diameter) were applied as precursors for the preparation of composite fibers as well as composite films. The CNC–CoFe_2_O_4_ composite fibers showed ferromagnetic properties, having a saturation magnetization of ~20 emu g^−1^. The potential application of precursor dispersions was demonstrated, and possible applications of the studied CNC-based magneto-responsive materials in the biomedical and magneto-optical components were highlighted such as magnetic shielding, packaging, separation applications, cell treatment, and drug delivery [175]. 

The number of publications on cellulose supported/templated inorganic oxide nanostructures is also extensive and therefore not all of them can be included in the text of the review. Consequently, the information on the inorganic oxides supported by the cellulose structures is collected in Table 3. The most commonly used method of metal oxide deposition on the cellulose is the adsorption of metallic ions via hydroxyl groups, followed by the formation of oxide particles by a hydrothermal or solvothermal reaction, or by a sol–gel process. Alternative methods of deposition involve a separate formation of nanoparticles, followed by their deposition onto the cellulose surface or commercial powders as well as various physical methods such as laser vaporization or ultrasonication, etc. The types of cellulose substrate range from natural cellulose fibers (CF) via microcrystalline cellulose (MCC) or microfibrillated cellulose (MFC) to bacterial nanocellulose (BNC) and cellulose nanocrystals (CNCs). The most common applications of these structures range from various (photo)catalytic systems to antibacterial agents in medicine and as adsorbents for drinking and wastewater purification.

## 4. Cellulose Templated Pure Metal Oxide Nanostructures

The sol–gel mineralization of cellulose nanofibers is a flexible way to produce inorganic frameworks of a controlled size, structure, and porosity. For example, hollow nano-objects have raised interest in applications such as sensing, encapsulation, and drug-release [238]. A porous inorganic structure template by the cellulose structures can be prepared by the formation of metallic oxides on the surface of the cellulose and subsequent calcination. In some cases, the solvothermal removal of the cellulose template is also used [239]. Various porous structures of a wide range of pure metal oxides have been reported to be prepared by chemical or physical methods using different cellulose structures as templates. Porosity significantly increases the specific surface of these materials, giving these materials a high potential in catalysis, photovoltaics, sensorics, electrochemical energy storage, etc. [15]. The tunable porosity of inorganic oxide thin films is a key factor for their successful applications in photovoltaics, sensing, and photocatalysis. Direct cellulose templating of inorganic oxides has been carried out with nanocellulose aerogels, microfibrillated cellulose, bacterial cellulose, nanocrystalline cellulose, filter paper, and green leaves. It is expected that the pore size is a function of the shape and dimensions of the applied cellulose template. The resulting materials were sponge-like inorganic oxide monoliths, freestanding films, or powders. Additionally, titania powders biotemplated with cotton fibers were further processed to a paste that was afterward coated on conducting substrates and applied in a solar-cell assembly.

**Scheme 3:**
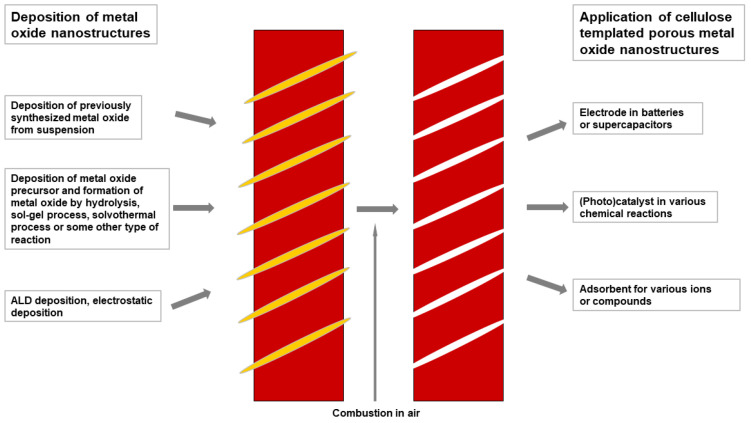
The schematic representation of the cellulose/metal oxide nanocomposites and porous metal oxide structures, synthetic routes, and the most frequent applications of these materials.

**Scheme 4:**
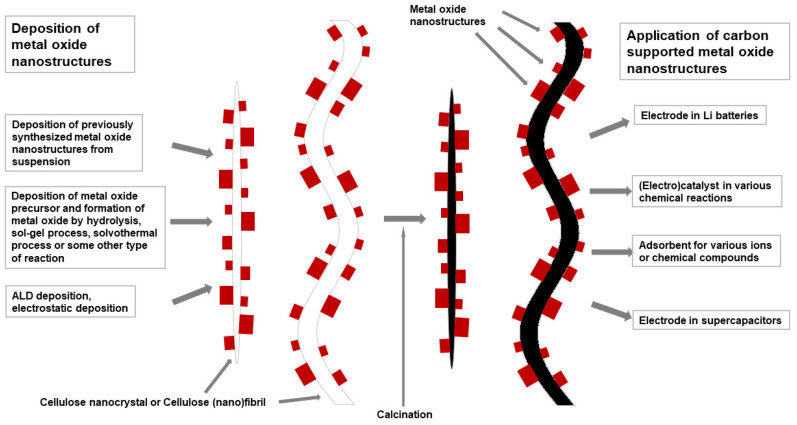
The schematic representation of the carbon fiber supported metal oxide nanostructures, synthetic routes, and most frequent applications of such materials.

Cellulose in all of its structures and variations proved to be a highly suitable sacrificial biotemplate for the synthesis and formation of porous and mesoporous hierarchical structures or aerogels of the whole variety of inorganic oxides. All types of cellulose have been applied as biotemplates for the porous structures of inorganic oxides. The list includes TiO_2_, ZnO, Fe_2_O_3_, CuO, Mn_3_O_4_, Nb_2_O_5_, Co_3_O_4_, SnO_2_, and more complex mixed oxides such as indium tin oxide, Cu_0.5_Co_0.5_Fe_2_O_4_, or BaFe_12_O_19_/CoFe_2_O_4_. Unfortunately, there are very few reviews based solely on cellulose-templated porous pure inorganic materials [240]. The most frequently studied cellulose template porous material is TiO_2_, which is a very promising catalyst in the photoelectochemical water splitting of cells [241]. Porous materials are also important in the sorption of gases such as CO_2_, which is highly important for the reduction in the CO_2_ concentration in the environment [242]. Recently, the last area of application is gaining significant importance since the number of electrically driven vehicles is rapidly increasing [243]. Cellulose, as an environmentally-friendly shape persistent biotemplate, offers the possibility of the formation of new shapes and forms of inorganic oxides with tunable porosity through mostly simple and environmentally-friendly procedures followed by calcination. Cellulose is an ideal organic support and sacrificial template since it contains a lot of oxygen, and upon calcination in air, it completely degrades, leaving pure inorganic compounds with high porosity. If the calcination is performed in an inert atmosphere, carbon-supported or carbon-doped materials are formed [240]. Aside from a high specific surface, the density of the prepared material was significantly reduced, and in many cases, special structures were formed such as hollow 1-D fibers, microrods, nanowires, or nanotubes. The capability of the cellulose nanocrystals to organize into lyotropic liquid crystalline structures was exploited in the formation of nematic mesoporous silica films that were further applied as templates for inorganic oxides organized into photonic crystals with periodic order and tunable optical properties [244]. In addition to classical chemical synthesis, other innovative processes have also been applied such as the sonochemistry process, microwave-assisted ionic liquid process, evaporation-induced self-assembly, and atomic layer deposition method. In Scheme 3, the versatility of cellulose substrates, the formation processes of metal oxide porous nanostructures, and the potential applications of these structures are presented. The versatility of the cellulose substrates, the formation processes of carbon fibers supported metal oxide nanostructures, and the potential applications of these hierarchical structures is presented in Scheme 4. 

### 4.1. Cellulose Templated Porous TiO_2_

**Figure 9:**
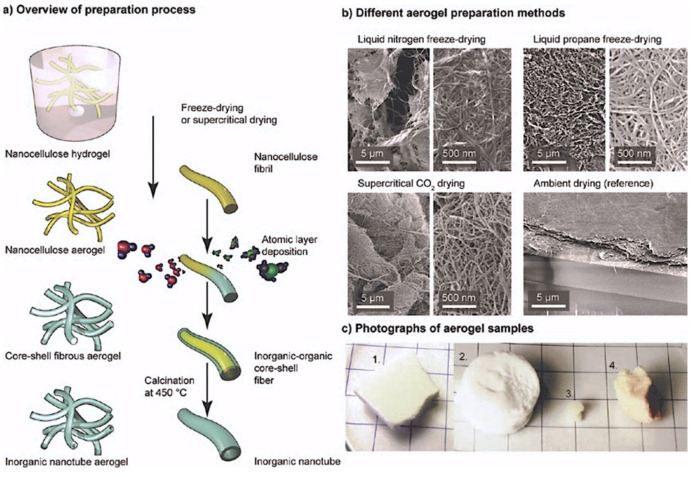
(**a**) The schematic presentation of the inorganic hollow nanotube preparation process. First the nanocellulose hydrogel is dried to obtain the aerogel, which is then coated with inorganic oxides using the ALD method to form composite organic/inorganic nanofibers. Finally, the composite was calcined to inorganic hollow nanotubes. (**b**) The SEM images demonstrating the effect of different drying methods: freeze-drying by freezing the nanocellulose hydrogel in liquid nitrogen, followed by the sublimation of ice in vacuum, leads to aerogels with sheet-like aggregates; freeze-drying in liquid propane leads to fibrillar aerogels with suppressed aggregation, given the sample is sufficiently thin; supercritical-drying leads to fibrillar aerogels without aggregates, even in thick samples; drying at ambient conditions leads to the collapse of the structure. (**c**) Photographs of the aerogel samples: (1) Liquid propane freeze-dried aerogel of 2 mm thickness; (2) supercritically-dried sample with ca. 12 mm in diameter and 10 mm in height; (3) atmospherically-dried sample that completely collapsed. Wet dimensions were the same as in the supercritically-dried sample; (4) Supercritically-dried aerogel after ALD showed a slight yellow color on the surface. Reprinted with permission from [238]. Copyright 2011 American Chemical Society.

This section addresses the formation of (nano)porous TiO_2_ structures using any type of cellulose as a sacrificial template. The formation of nanotube aerogels with a framework composed of inorganic hollow nanotubes was reported. A preparation route of TiO_2_, Al_2_O_3_, and ZnO nanotube aerogels by applying biological aerogel templates such as microfibrillated cellulose (MFC) or cellulose nanocrystals (CNC) (Figure 9) using the atomic layer deposition method (ALD) was demonstrated. Freeze-drying or supercritical-drying was used for the preparation of aerogel templates as freestanding objects or films on substrates from nanocellulose hydrogels. Supercritical-drying produced nanocellulose aerogels without major interfibrillar aggregation, even in thick films. Uniform oxide layers were readily formed by ALD onto the fibrils, producing organic–inorganic core-shell nanofibers. The cellulose core was removed by calcination at 450 °C, forming inorganic self-supporting aerogels containing hollow nanotubular networks. They were also dispersed in ethanol by grinding to prepare a hollow nanotube slurry, which was cast to form a porous inorganic film. The TiO_2_ nanotube network was tested as a fast response humidity sensor [238].

**Figure 10:**
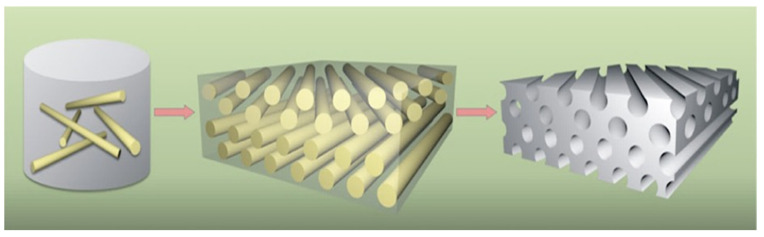
The synthesis approach to the NCC-templated mesoporous TiO_2_ film. Reprinted with permission from [15]. Copyright 2014 American Chemical Society.

Cellulose nanocrystals (CNCs) were applied as a shape-persistent template allowing for the straightforward formation of mesoporous TiO_2_ films (Figure 10). The formed TiO_2_ structures were high porosity anatase films with narrow pore size distributions and well-defined pores. By varying the template to the TiO_2_ ratio, the pore size, pore anisotropy, surface area, and diameter of the titania nanoparticles in the films were controlled. Then, by applying a post-treatment at high humidity followed by slow template removal, the pore widening was achieved and this procedure was useful for the multilayer deposition to prepare thick films. Homogeneous and transparent films were prepared by the spin- or dip-coating on various substrates such as glass, silicon, and transparent conducting oxide (TCO). The formed mesoporous TiO_2_ films exhibited high activity in the degradation of 4-chlorophenol as well as in the photocatalytic conversion of nitrogen oxide and they were successfully applied as anodes in dye-sensitized solar cells. The results revealed that CNC-templating offered the potential to supersede conventional processes toward porous TiO_2_ and that this process showed a potential for application in the preparation of mesoporous films with other inorganic oxides [15].

Mo-doped titania thin films with high transparency were formed on an ITO glass substrate by spin-coating the titania composite precursor, which was prepared by mixing titanium(IV) ethoxide and HCl, followed by the addition of CNCs and molybdenum(V) chloride. Subsequently, the prepared composite film was calcined at 400 °C for 1 h to obtain an ITO supported titania porous film. Due to the presence of CNCs as a sacrificial template, deposited titania films, composed of TiO_2_ crystals with average particle sizes of 10 nm, contained slit-like pore channels with a pore width ranging from 5.7 to 7.8 nm and a surface area of 150 m^2^ g^−1^. The photocatalytic properties of the formed films were studied for the degradation of trichloro ethylene (TCE) relative to the non-templated solid titania film as well as to the undoped CNC-templated porous titania film. This evaluation showed that both the CNC-templating and the Mo-doping significantly increased the photocatalytic activity of the TiO_2_ films for TCE degradation. Additionally, the Mo-doped TiO_2_ films exhibited very strong visible-light absorption and are expected to have high potential as sustainable coatings for photocatalysis and solar cells. The reported synthesis method of TiO_2_ with the application of CNCs as a sacrificial template was demonstrated to be a low-cost, simple, and energy saving process to fabricate highly transparent and mesoporous thin photocatalytic films, offering a significant enhancement in the photocatalytic performance with a two-fold faster degradation of TCE [245].

Recently, a new class of mesoporous materials with a chiral nematic pore structure templated by the lyotropic liquid-crystalline phase of cellulose nanocrystals (CNCs) was developed. The condensation of Si(OMe)_4_ as a silica precursor in the presence of CNCs produced a composite material in which CNC particles with a nematic chiral organization were embedded in the SiO_2_ matrix. The helical pitch in the mesoporous SiO_2_ was tuned by varying the ratio of the CNC to Si(OMe)_4_, thus changing the reflected light wavelength in the range from the near IR to the UV. After the removal of the CNC with 12 M HCl at 85 °C followed by a short oxidizing treatment with H_2_SO_4_/H_2_O_2_ (5:1), porous SiO_2_ was obtained as a freestanding film with a long-range chiral nematic structure. The mesoporous anatase TiO_2_ was synthesized from TiCl_4_ by calcination at 600 °C by using a nematic chiral mesoporous SiO_2_ film, acting as a hard template, which was subsequently removed by etching with 2 M NaOH to prepare a free-standing structure of the mesoporous TiO_2_ with a specific surface area between 150 and 230 m^2^ g^−1^. The structure of the chiral nematic SiO_2_, originating from the lyotropic liquid crystalline ordering of CNC, was preserved in the final product, producing a chiral nematic mesoporous TiO_2_ film, reflecting the circularly polarized light. This study is the original approach toward organizing a crystalline metal oxide into a chiral nematic structure. The reported synthetic method is highly attractive for preparing new catalytic and photonic materials [246].

### 4.2. Cellulose Templated Porous Iron Oxides

Cotton cellulose was applied as a sacrificial template in the in situ formation of porous and hollow CoFe_2_O_4_/BaFe_12_O_19_ microrods. The cellulose template was removed by calcination at 250 °C for 2 h and additionally at 900 °C for 3 h. The resulting composites possessed the porous hollow microrod morphology with soft ferrite (CoFe_2_O_4_) and hard ferrite (BaFe_12_O_19_) being uniformly deposited along the microrods. Due to the exchange–coupled interaction and their specific morphological effects, the microwave absorption characteristics of the microrods were substantially enhanced, as shown by electromagnetic studies. The microwave absorption bandwidth with the reflection loss less than −10 dB was expanded to 8.1 GHz, while at the same time, the weight content of the ferrite component in the composites was reduced to 60 wt.%. Therefore, the application of cellulose as a template proved to be an efficient approach to simultaneously reduce the mass of conventional ferrites and enhance the microwave absorption properties. The reported simple formation process of hollow porous CoFe_2_O_4_/BaFe_12_O_19_ microrods also increases the opportunities to develop a variety of soft/hard ferrite composite materials for other potential applications such as sensors, permanent magnets, and ultra-high-density data storage [247].

A simple method for the synthesis of crystalline and highly porous α-Fe_2_O_3_ thin films with various nanomorphologies has been developed by using the CNCs as a biotemplate. FeCl_3_, FeCl_2_, and Fe(NO_3_)_3_ were used as precursors in the CNC-templated synthesis. It was demonstrated that after the post-synthetic humidity treatment at 100 °C, the crystallization of Fe_2_O_3_ proceeded via the formation of β-FeOOH of a highly anisotropic shape. The porosity and homogeneity of the Fe_2_O_3_ films were preserved despite the complex crystallization behavior of Fe_2_O_3_ due to the shape persistence of CNCs. The calcination procedure of the CNC/Fe_2_O_3_ precursor composites showed a strong impact on the morphology and porosity of the Fe_2_O_3_ films. The calcination was performed at temperatures between 300 and 600 °C, depending on the type of precursor. It was demonstrated that a great variety of Fe_2_O_3_ networks could be synthesized and the morphology of their films could be tuned by simple templating with CNCs. The reported synthesis approach is advantageous for the formation of porous homogeneous Fe_2_O_3_ films on various kinds of substrates [248].

### 4.3. Miscellaneous Cellulose Templated Porous Metallic Oxides

Natural cellulosic fibers (kapok fibers) were applied as sacrificial templates in the formation of hollow SnO_2_ nanotubes by applying the surface sol–gel process followed by calcination at 550 °C for 5 h. The prepared nanostructures, consisting of SnO_2_ nanoparticles being smaller than 12 nm in size, were hollow nanotubes with a diameter between 50 and 100 nm, while the thickness of the tube wall was estimated to be from 10 to 20 nm. The resulting SnO_2_ nanotubes showed a high BET surface area of 185.15 m^2^ g^−1^ and very high aspect ratios. The coulombic efficiencies larger than 97.8% and stable discharge capacities of ~600 mA h g^−1^ were measured by using the current density of 100 mA g^−1^. The characteristics of the nanostructured SnO_2_ particles embedded in the nanotube resulted in high electronic conductivity and high capacity of the electrode. The hollow structure provided enough void space so that the SnO_2_ nanoparticles changed a volume without a collapse, causing excellent cycling stability of the electrode. This preparation approach is low-cost, facile, and is suitable for the scale-up production of nanotubular materials that are applicable in gas sensors or in lithium-ion batteries [249].

**Figure 11:**
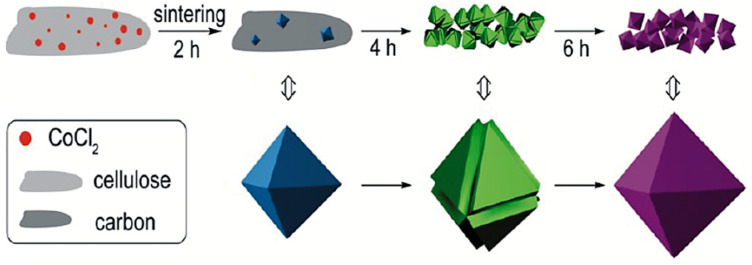
The schematic illustration describing a time-dependent growth of Co_3_O_4_. Reprinted with permission from [250]. Copyright 2014 American Chemical Society.

Cellulose was applied as a template in the preparation of octahedral, face-raised Co_3_O_4_ crystals to be applied as a catalyst in the selective oxidation of alcohols. Initially, the CoCl_2_ precursor and cellulose were mixed in solution, dried, and calcined at 500 °C for various times of 2–6 h. The edge length of the prepared Co_3_O_4_ structures was about 200 nm and they formed microtubes with a width of 2–3 μm and a length of ca. 10 μm. The formation of the Co_3_O_4_ octahedral structure and assembly process were facilitated by the thermal decomposition and carbonization of cellulose (Figure 11). The face-raised Co_3_O_4_ octahedral nanocrystals showed higher saturation magnetization and remanence due to smaller grain sizes with higher uniformity compared to the normal Co_3_O_4_ octahedra. Additionally, the Co_3_O_4_ octahedra grown by the cellulose-assisted process exhibited both the higher selectivity and higher activity in the catalytic oxidation of alcohols compared to the normal Co_3_O_4_ octahedra. For example, the reported Co_3_O_4_ nanocrystals showed a very high catalytic activity after eight cycles, giving them a high potential in heterocatalytical applications [250].

The cellulose/CuO nanocomposites were prepared by an ionic liquid microwave-assisted process from the microcrystalline cellulose solution, CuCl_2_ precursor, and BmimBF_4_ medium. The reported procedure combined three highly important green chemistry principles: sustainable resources, green solvents, and an environmentally-friendly method. With longer microwave-heating time, the crystallinity of CuO increased and the CuO shape changed from nanosheets to bundles composed of irregular nanosheets, and finally, to regular nanoparticles. The ratio of the cellulose solution to ionic liquid significantly affected the microstructure and morphology of CuO in the nanocomposites. The pure CuO structures with various morphologies and particle sizes were obtained by the calcination of different precursors (cellulose/CuO nanocomposites) at 800 °C for 3 h. The reported synthetic approach opens a novel path toward the formation of inorganic oxides and to the application of cellulose with a high-added value [251].

Pure ZnO nanoparticles with a wurtzite structure have been prepared by using bacterial cellulose (BC) embedded with zinc acetate as a template and by subsequent calcination. The morphology and average particle size of ZnO nanoparticles were tuned by varying the calcination temperature and the concentration of zinc acetate in an aqueous solution. The presence of BC strongly influenced the size, morphology, and size distribution of the formed ZnO. Highly crystalline ZnO nanostructures with a narrow particle size distribution from 20 to 50 nm and high photocatalytic activity were prepared by using BC as a template containing 1 wt.% zinc acetate and by calcination at 600 °C for 2 h. The reported process provided an efficient, facile as well as environmentally-friendly approach toward the formation of the highly defined and homogeneously dispersed ZnO nanostructures. The reported process can be applied for the preparation of other nanoscaled metal oxide particles. The only disadvantage of this approach is the rather high calcination temperature, which can cause sintering of the ZnO nanoparticles [252].

**Figure 12:**
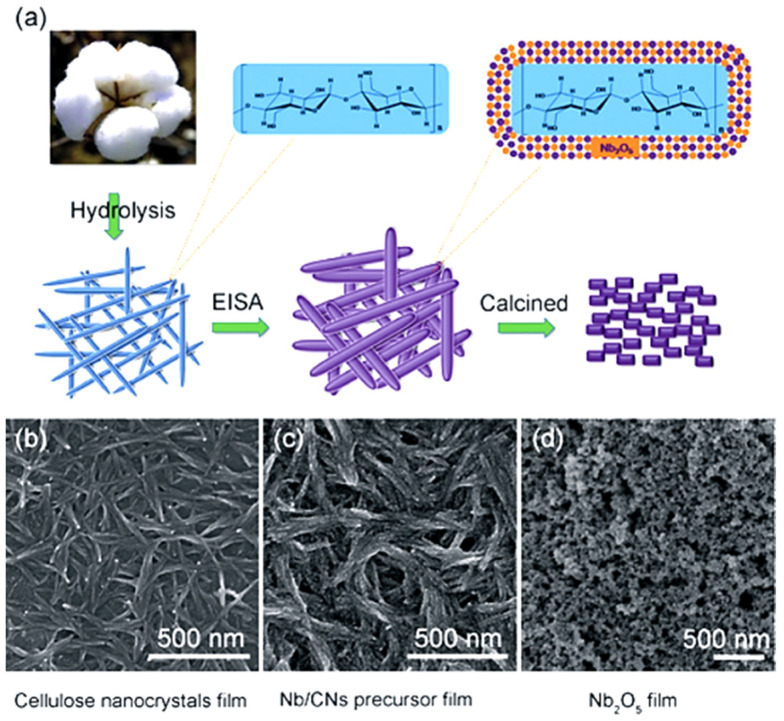
The schematic presentation of the Nb_2_O_5_ nanostructures prepared by an evaporation-induced self-assembly process directed by CNCs (**a**), the SEM micrographs of the CN film (**b**), the CN/NbCl_5_ composite film (**c**), and the mesoporous film of Nb_2_O_5_ after calcination (**d**). Reprinted from [253]. Copyright 2014, with permission from Elsevier B.V.

Inspired by the unique structural and surface characteristics of cellulose nanocrystals (CNCs), Kong et al. used an evaporation-induced self-assembly process to prepare niobium oxide/CNC composite films (Figure 12) [253]. After the calcination of the CNCs in air at 600 °C, mesoporous *T*-Nb_2_O_5_ films were obtained that were composed of interconnected nanocrystals (size ~50 nm). By varying the NbCl_5_/CNC mass ratio, it was possible to control the crystallite dimensions (20–50 nm) and the surface area of the Nb_2_O_5_ nanostructures. When studied as the electrode materials, these *T*-Nb_2_O_5_ nanocrystals showed very high Li^+^ intercalation pseudo capacitance, wide temperature adaptability, and excellent cycle performance. The superior charge storage and kinetics benefits of the prepared *T*-Nb_2_O_5_ nanocrystals enabled an innovative approach toward the design of materials providing high energy density and high power density at the same time for electrochemical energy storage applications. 

Europium (Eu^3+^) doped, chiral nematic, mesoporous luminescent films of ZrO_2_ (EDCNMZrO_2_) with tunable optical properties were prepared by a hard template process using silica templated by cellulose nanocrystals (CNCs). Chiral nematic SiO_2_ films were formed by replicating the chiral nematic structure of CNC using an evaporation-induced self-assembly (EISA) approach, followed by calcination at 600 °C for 6 h, and subsequent silica etching. The obtained EDCNMZrO_2_ showed selective suppression of the spontaneous emission of Eu^3+^ ions as well as the possibility of modulating their luminescence lifetime. This study is a showcase of how the capability of CNC to organize into the liquid crystal nematic phase can be exploited as an indirect template to prepare the inorganic (ZrO_2_) photonic material, which has potential application in novel smart optical nanodevices [254]. 

### 4.4. Inorganic Nanostructures Supported by Carbon Fibers Formed from Cellulose

**Figure 13:**
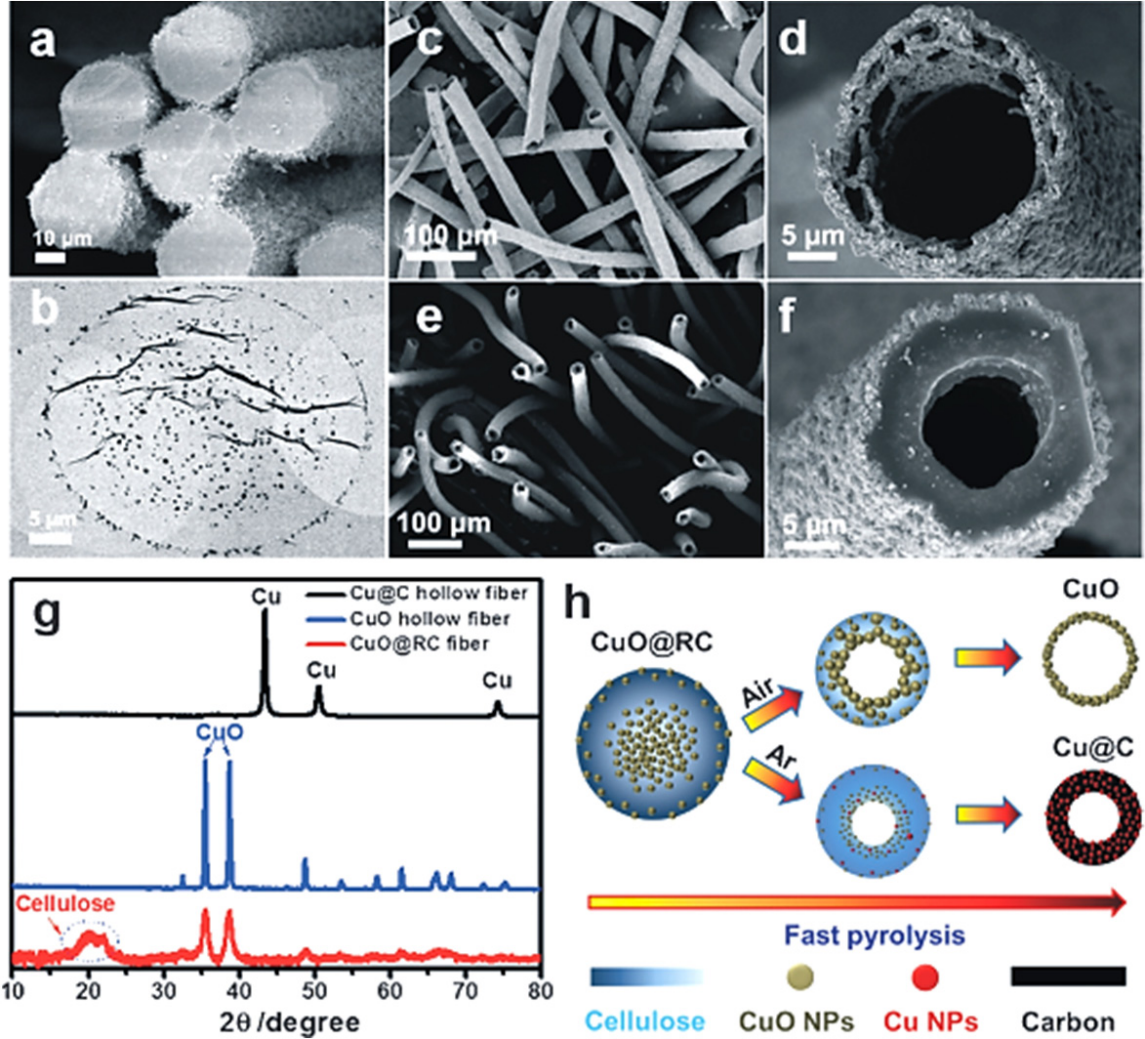
(**a**) The SEM and (**b**) TEM images of the CuO–RC filaments. (**c**,**d**) SEM images of CuO-and (**e**,**f**) SEM images of the Cu@C hollow fibers. (**g**) The XRD patterns of the CuO–RC fibers, CuO, and Cu–C hollow fibers. (**h**) The formation of CuO and Cu–C hollow fibers by the fast pyrolysis of CuO–RC filaments. CuO and Cu–C hollow fibers were obtained by the pyrolysis of CuO–RC filaments at 600 °C for 1 h in an air and argon atmosphere, respectively [259]. Copyright 2016 John Wiley & Sons Inc. Reproduced with permission.

A special case represents the carbon nanofibers formed from various cellulose structures by pyrolysis in an inert atmosphere. Such structures are thermally stable and electrically conductive. When bacterial nanocellulose is used, templated carbon networks are obtained. Depending on the raw cellulose material, a whole variety of porous carbon nanostructures can be formed. For example, when wood was first delignified, the resulting cellulose hierarchical structure was exposed to cellulose enzymes to partially degrade its structure to form micro- and nanopores, followed by carbonation at 1000 °C for 3 h in a N_2_ atmosphere, a highly porous hierarchical carbon structure was obtained. Such structures with a high specific surface have been applied as electrodes in supercapacitors with high areal/volumetric energy density and excellent stability [255]. Porous carbon hierarchical structures based on wood cellulose are frequently studied as electrodes in supercapacitors or in batteries [256,257]. Various cellulose raw materials including agro waste can be applied to form porous hierarchical carbon structures [258]. Carbonized cellulose structures have a wide range of potential applications. For example, regenerated cellulose (RC) fibers with low crystallinity have been used as a template for the formation of inorganic hollow structural materials by a simple pyrolysis procedure. The RC–CuO composite fibers were composed of cellulose fibers and a high concentration of CuO NPs inside the fibers. The obtained heterogeneous system allowed for the specific thermal decomposition mechanism of the cellulose template during the pyrolysis process, involving the migration of the CuO NPs from the center to the fibers’ edge (Figure 13). At the end, Cu@C and CuO hollow microfibers with a controllable morphology and size were formed in an argon and air atmosphere, respectively. Aside from the copper-based hollow structures, other metallic hollow structural materials such as the Ni@C as well as the hollow structures of many inorganic oxides (NiO, Fe_2_O_3_, Mn_2_O_3_, Fe_3_O_4_@C, MnO@C) have been prepared through this process. The reported approach to create hollow fibers was demonstrated to be scaled-up easily and the application of elaborated processes as well as expensive starting materials was avoided. The potential applications of the prepared hollow inorganic fibrous materials are in microfluidics, as microreactors, and in catalysis [259].

Nitrogen-doped carbon networks were formed by carbonizing the polyaniline (PANI) coated BNC at 850 °C for 2 h in a flow of N_2_ where the bacterial nanocellulose (BNC) was applied as the precursor and template. The obtained carbon networks functioned as conductive networks for the integration of active electrode materials providing the fast electron transfer and as a support for the preparation of high capacitance electrode materials. MnO_2_ was formed on the surface of the activated carbon network by the redox reaction using KMnO_4_ as a precursor. The prepared asymmetric supercapacitors, based on activated carbon/carbon-MnO_2_, showed a maximum power density of 227 kW kg^−1^ and a high energy density of 63 W h kg^−1^ in an aqueous solution of 1.0 M Na_2_SO_4_. Their energy density was higher than most values reported for the activated carbon//MnO_2_ asymmetric supercapacitors. Additionally, the resulting supercapacitor also showed a formidable cycling performance with a 92% specific capacitance retention after 5000 cycles. The reported approach represents an environmentally-friendly and low-cost preparation process of electrode materials with potential application in high-performance energy storage devices [260].

The thermal reduction of metallic nanoparticles in the presence of cellulose is questionable due to its rather low thermal stability, but the thermal reduction of metals in an inert atmosphere accompanied with carbonization of the cellulose is possible. For example, metallic Ni nanoparticles deposited on bacterial cellulose-derived carbon fibers (Ni-NPs/BCCF) were synthesized using a simple impregnation-pyrolysis method as efficient electrocatalysts for a two electron oxygen reduction reaction (2e^−^ ORR). NiCl_2_ was first deposited on BC by its immersion in an aqueous solution of the precursor (NiCl_2_-conc. = 0.1 mol L^−1^) for 24 h and subsequently freeze-dried. The resulting material was pyrolyzed first at 360 °C for 2 h and then at 700 °C for an additional 2h both in an Ar atmosphere. After cooling down to RT, the solid product was ground in a mortar, washed several times with deionized water and ethanol, and dried for further use. In 0.1 mol L^−1^ KOH, the Ni-NPs/BCCF-20.7 sample showed a high electrocatalytic performance toward 2e^−^ ORR, exhibiting a H_2_O_2_ selectivity of ~90% and an onset potential of 0.75 V vs. the reversible hydrogen electrode (RHE). By applying this catalyst, a high Faradaic efficiency (93.9% ± 4.2% at 0.5 V vs. RHE in bulk ORR electrolysis) and a high H_2_O_2_ yield rate (162.7 ± 13.7 mmol g_cat_^−1^ h^−1^ at 0.2 V vs. RHE) were achieved. This work provides new insights into the design and development of high efficiency 2e^−^ ORR electrocatalysts for H_2_O_2_ production [261].

The number of publications on cellulose-templated or cellulose-based carbon fiber supported inorganic structures is quite numerous and they are summarized in Table 4. Table 4 itself is divided into two parts: the first part is devoted to the publications on cellulose-templated porous purely inorganic structures, while the second part comprises the publications on inorganic nanostructures supported by cellulose-based carbon (nano)fibers. The preparation procedures in both cases were similar; that is, the formation of inorganic structures templated by cellulose, followed by thermal treatment of the structure. The difference was only in the type of atmosphere used during the thermal processing. In the first case, the thermal treatment was performed in air, causing the complete degradation of the cellulose structure and leaving predominantly porous inorganic structures, while in the second case, thermal treatment was carried out in an inert atmosphere, causing the carbonization of cellulose and producing carbon fiber supported inorganic structures. The resulting structures were predominantly tested for (photo)catalytical systems, anodes in Li batteries, or electrodes in various sensors.

## 5. Cellulose Supported/Templated Nanostructures of Metal Sulfides, Hydroxyapatites, and Other Inorganic Compounds

**Scheme 5:**
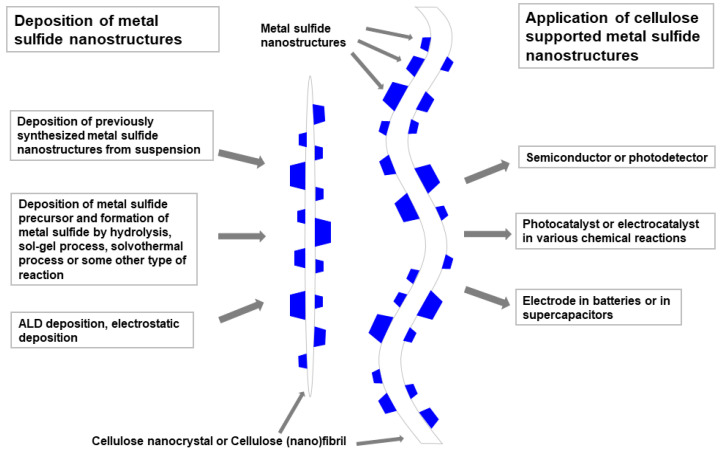
The schematic representation of the cellulose/metal sulfide particle nanocomposites, synthetic routes, and the most frequent applications of such materials.

The synthesis of cellulose/inorganic nanohybrid materials has become an important topic of research in science and industry since it offers novel and innovative applications in catalysis, energy harvesting, energy conversion and storage, drug delivery, etc. Aside from metallic and metal oxide particles, other inorganic compounds such as various inorganic sulfides, apatites, halides, etc. can be deposited or formed by using cellulose as a support and/or template. Other than metals and metal oxides, cellulose-templated hierarchical structures of other types of inorganic compounds can also be prepared such as various sulfides, halides, carbonates, and phosphates. The formation of cellulose supported metallic sulfides has not been as intensely studied as metals and metal oxides, but there has been a review partially dealing with this topic [297]. Deposition of AgCl on the bacterial cellulose is a simple route to the manufacturing of hydrophilic antimicrobial membranes, which are a promising alternative for antimicrobial wound dressings. Cellulose has been proven to be an alternative universal platform for engineering a variety of functional materials at the nanoscale. Cellulose hydroxyl groups not only anchor metallic ions tightly on the BC nanofibers via the ion–dipole interaction, but also stabilize inorganic nanoparticles by a strong interaction with their surface hydroxyl groups, thus preventing their aggregation. In many cases, the authors have used cationic surfactants to facilitate the synthesis of well-defined semiconductor nanoparticles on the cellulose surface. A special case is represented by decorated cellulose-based materials or surface-coated with carbonates, and especially with various types of hydroxyapatites. The resulting inorganic cellulose-based composites are biocompatible and capable of facilitating the osteoblast cell attachment and growth. Such materials have potential application as bone tissue scaffolds and in other biomedical fields. Additionally, these materials exhibit an effective adsorption of various ions from water solutions. Consequently, they are promising water purification materials distinguished by fast adsorption rates and high capacities. The presentation of the versatility of cellulose substrates, the formation processes of the metal sulfide nanostructures, and the potential applications of these hierarchical structures are shown in Scheme 5. 

**Scheme 6:**
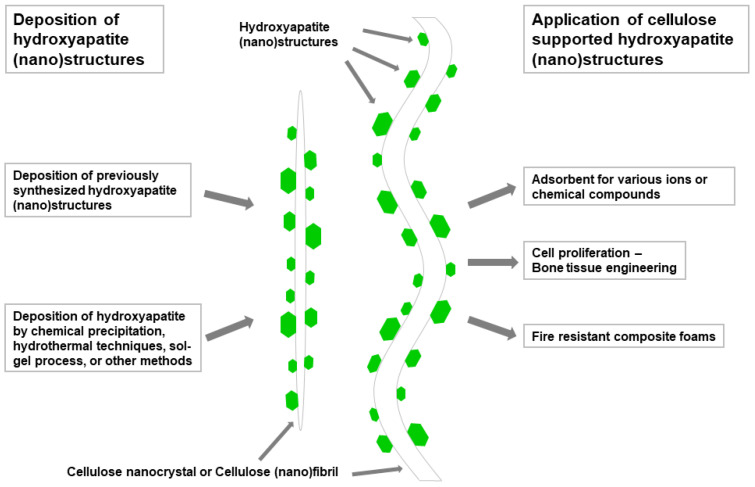
The schematic representation of the cellulose/hydroxyapatite nanocomposites, synthetic routes, and the most frequent applications of these materials.

In Scheme 6, the versatility of the cellulose substrates, the formation processes of hydroxyapatite nanostructures, and the possible applications of these hierarchical structures are presented. 

### 5.1. Cellulose Supported/Templated Metal Sulfides

By using the porous structure of the regenerated cellulose, the CdS nanostructures were formed from the CdCl_2_ solution. The cellulose pores functioned as reaction cavities where the CdS nanoparticles were grown and deposited in the shell composed of the cellulose fibers that prevented the aggregation of forming nanostructures. The CdS nanoparticles (size 6 nm) were homogeneously distributed and tightly adsorbed onto the cellulose structure by the strong intermolecular interaction between the CdS and cellulose fibers, thus preventing their aggregation. The CdS/cellulose composite films showed a substantially enhanced photocatalytic activity due to the porous structure of the cellulose template and the presence of the CdS nanoparticles. Additionally, for the promotion of H_2_ evolution, Pt was applied as a cocatalyst. This was formed in situ on the surface of the CdS–cellulose films by photo deposition using H_2_PtCl_6_ as a precursor. The cellulose fiber supported CdS nanoparticles showed a high efficiency in the photocatalytic evolution of hydrogen, and as a portable photocatalyst, they were easily recovered, avoiding environmental pollution [298]. 

An innovative nanocomposite aerogel composed of TEMPO oxidized microfibrillated cellulose embedded in a layer of previously synthesized ultrathin, fire retardant nanosheets of MoS_2_ was prepared. The cross linking between Mo^4+^ cations and carboxyl (–COOH) as well as hydroxyl (–OH) groups on the cellulose surface caused intense interaction between the MoS_2_ nanosheets and the cellulose fibers, thus enabling the formation of an aerogel with a density of 0.00473 g cm^−3^ and a 97.36% porosity. The resulting material showed outstanding self-extinguishing capabilities and fire resistant properties as characterized by cone calorimetry as well as by the vertical burning test. The MoS_2_ structure in the aerogel remained unchanged after the vertical burning test, as shown by Raman spectroscopy. This study represents a new approach toward the preparation of fire resistant and lightweight aerogel materials for application in the construction of fire resistant and energy efficient buildings as well as aerospace applications [299].

Various semiconducting sulfide nanostructures such as ZnS, PbS, and CdS have been prepared by using cellulose nanocrystals (CNCs) as supports combined with cationic surfactants as stabilizers. Their synthesis on the CNC surface was carried out by reacting precursor salts such as ZnCl_2_, Pb(NO_3_)_2_, and CdCl_2_ with H_2_S gas. In the controlled growth of defined semiconductor nanoparticles, the application of a cetyltrimethylammonium bromide (CTAB) cationic surfactant was the crucial factor. The pH of the salt solution and precursor concentration were varied to control the packing density and average particle size of the sulfide semiconductor nanostructures on the CNC surface. This procedure could serve as an innovative universal platform for engineering a variety of nanoscale functional materials for optical and electronic nanodevices [300].

### 5.2. Cellulose Supported/Templated Hydroxyapatites

Carbonated hydroxyapatite (CHA) modified microfibrillated cellulose (MFC) was studied as an adsorption substrate for Ni^2+^, Cd^2+^, PO_4_^3−^, and NO_3_^−^ ions from the aqueous solution. The MFC was initially treated with NaOH and CHA was subsequently deposited on its surface by the reaction of CaCl_2_ and NaH_2_PO_4_. The obtained results revealed that the removal of all of the studied ions was not dependent on the pH and was highly effective for Ni^2+^, Cd^2+^, and PO_4_^3−^. Namely, the adsorption efficiency for NO_3_^−^ removal from an aqueous solution reached more than 50% while the adsorption efficiencies for the other three ions were higher than 90%. The highest observed removal capacities of the studied CHA/MFC adsorbent for the NO_3_^−^, PO_4_^3−^, Cd^2+^, and Ni^2+^ ions were 0.209, 0.843, 1.224, and 2.021 mmol g^−1^, respectively. The adsorption kinetics was very fast for all of the studied ions. This study demonstrated that CHA/MFC was an environmentally-friendly and practical absorbent, exhibiting high removal efficiencies for both the metallic and phosphate ions while the removal efficiency for the nitrate ions was only moderate [301]. 

A biomimetic approach was applied for the development of hydroxyapatite/bacterial cellulose nanocomposites with bone healing properties. The nucleation of calcium-deficient hydroxyapatite (cdHAp) was initiated by the adsorption of carboxymethyl cellulose (CMC) on two forms of bacterial cellulose (BC) (pellicles and tubes). The dynamic simulated body fluid (SBF) was used in the formation of the cdHAp in vitro over a one-week period. The atomic content of phosphorus and calcium varied from 0.3 to 11.2 atomic % and 0.4 to 7.7 atomic %, respectively, while the Ca/P overall ratio changed from 1.2 to 1.9 and the crystal size of cdHAp decreased with the decrease in the density of bacterial nanocellulose fibrils. The differentiation and morphology of the osteoprogenitor cells were studied through alkaline phosphatase gene expression and fluorescence microscopy to determine the viability of the scaffolds in vitro. The BC surface coated with cdHAp crystals showed an enhanced cell attachment, indicating that the BC-cdHAp scaffolds were a feasible and promising bone scaffolding material [302].

Cellulose (BC) is a high-strength fibrous and extensively used biomaterial while hydroxyapatite (HAp) has outstanding osteoconductivity and bioactivity. Composite materials composed of cellulose matrices and inorganic nano-hydroxyapatite (n-HA) fillers have received considerable attention in bone engineering, although the brittleness and low strengths limit their application. Therefore, the improvements in the toughness and mechanical strength of aerogel-based biomaterials are necessary. A urea alkali system was applied to disperse and gelate cellulose mixed with silk fibroin (SF) to prepare the composite aerosol. n-HA was prepared by swelling PAA with water for 20 h and then CaCl_2_ was added and stirred at 90 °C until complete dissolution. Subsequently, the Na_2_HPO_4_ solution (Ca/P = 1.67) was admixed into the prepared solution with the pH adjusted to 10 using a NaOH solution. After this, the solution was stirred for 2 h at 90 °C followed by aging at RT for 24 h. At the end, the resulting precipitate was freeze-dried and calcined at 800 °C to prepare the n-HA. By using uniaxial compression, the composite aerogel material with a density close to that of natural bone was prepared with a high mechanical strength and toughness. When the ratio of the cellulose, n-HA, and SF was 8:1:1, the composite aerogel showed the same mechanical strength range as the cancellous bone. HEK-293T cells, cultured on these composite aerogels, exhibited a high ability of adhesion, differentiation and proliferation. The studied biodegradable composite aerogel has high potential to be applied as a bone tissue substitute in bone-tissue engineering [303].

**Figure 14:**
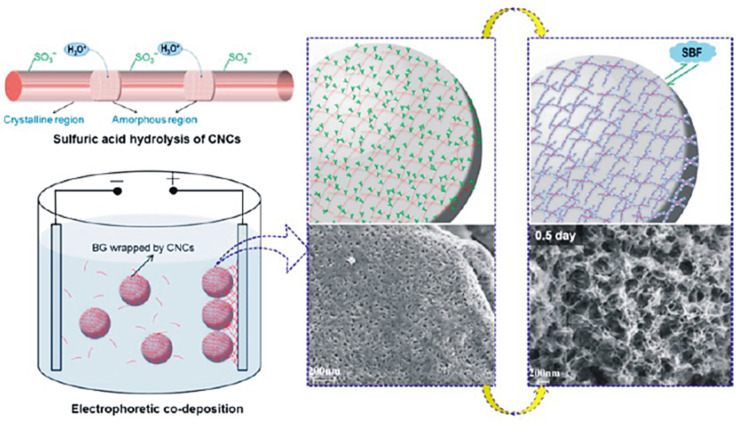
The schematic model describing the interaction between the BG particles and CNCs in the suspension, together with their electrophoretic co-deposition process as well as the mechanism of mineralization by the formation of HAp in the simulated body fluid. Reprinted with permission from [304]. Copyright 2015 American Chemical Society.

The one-step electrophoretic deposition process (EPD) was used for the preparation of an innovative bioglass—a cellulose nanocrystal (BG-CNC) bioactive hybrid material—to be applied for the surface coating of orthopedic implants with the purpose to enhance their contact with the bone tissue. The characterization of the prepared materials revealed that the network of CNCs functioned as an in situ template tailoring the mineralization rate and morphology of the formed hydroxyapatite (HA) crystal phase (Figure 14). The BG particles were individually wrapped by a nanoporous CNC layer. A very rapid formation of a HA layer (after 0.5 day) on the BG particles of the deposited BG-CNC coating in the simulated body fluid was observed during in vitro characterization. The cell culture tests using the MC3T3-E1 cell line revealed that the formed BG-CNC hybrid coatings represent a promising platform for the proliferation, accelerated attachment, and differentiation of cells, confirming their potential in biomedical applications as an advanced bone contacting material [304].

### 5.3. Miscellaneous Cellulose Supported/Templated Inorganic Compounds

Cellulose fibers with UV activity have been prepared by spinning of 8 wt.% α−cellulose solution in *N*-methylmorpholine-*N*-oxide (NMMO) using a dry–wet method and by subsequent surface modification of the fibers by the deposition of the Gd_4_O_3_F_6_:Eu^3+^ doped with 5 mol % of the Eu^3+^. During its dissolution process in NMMO, the photoluminescent nanostructures were admixed as a powder into the cellulose matrix. Photoluminescence spectroscopy was used to study the relationships between the emission intensity and the excitation energy as well as the concentration of Gd_4_O_3_F_6_:Eu^3+^ nanoparticles in the final composite products. About 80% of inorganic particles had a diameter below 300 nm when the samples contained up to 1% of the modifier, and with an increase in the modifier concentration, the agglomeration of the particles was intensified. The obtained cellulose fibers with UV activity have potential application as a security marking for documents, textile, and paper products [305].

Cobalt ferrite (CoFe_2_O_4_) nanostructures were formed on the surface of the cellulose structures from CoCl_2_ and FeSO_4_ as precursors in the presence of KNO_3_ and NaOH. These inorganic nanoparticles were applied as labels for fine cellulose fibers, representing an approach that allows for the detection of micro- and nanostructured celluloses in paper sheets in combination with various imaging technologies. The addition of nanostructured CoFe_2_O_4_ as a labeling material during the paper manufacturing process enables imaging in scanning electron microscopy/energy-dispersive X-ray spectroscopy experiments and provides contrast in X-ray microtomography. By combining these two techniques, the fines in the paper sheets were located, evaluated, and quantified. It was demonstrated that the water retention value, the tensile indices, and the air permeability of the handsheets were not changed by the addition of the CoFe_2_O_4_ labeled fines compared to the sheets where the nonlabeled fines were added. The knowledge about the distribution of nano- and microscale cellulosic structures in a cellulose fiber matrix is important to improve the properties of the existing paper products and in the future, the design of new materials in the paper industry [306].

The number of publications on sulfides, hydroxyapatites, carbonates, halides, and other types of inorganic materials is not as large as in the case of metallic oxides. These are summarized in Table 5. Metallic sulfides are mostly formed on the cellulose by hydrothermal or solvothermal processes using sodium sulfide, thiourea, or thioacetamide as a source of sulfur. Metallic sulfide–cellulose structures are predominantly used as photocatalysts, electrodes in super capacitors, and photoluminescent or fluorescent materials. Ca hydroxyapatites–cellulose structures are mostly prepared by the biological route from Na_2_H_2_PO_4_ or NH_4_H_2_PO_4_, and CaCl_2_ using simulated body fluid. These are used as substrates in bone tissue engineering or as absorbents for heavy metal ions.

## 6. Cellulose Supported or Templated Hybrid Inorganic Nanostructures

**Scheme 7:**
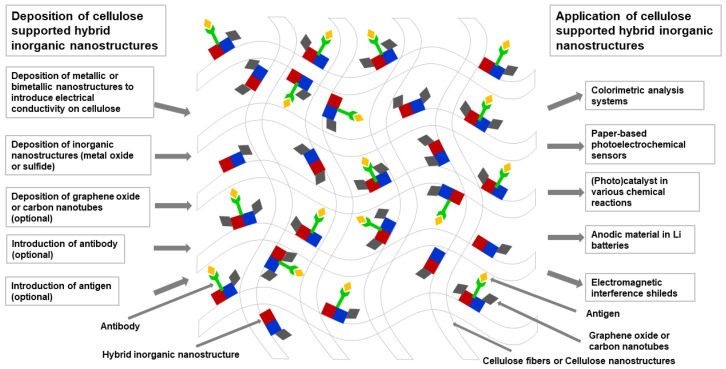
The schematic representation of hybrid cellulose/metallic or metal oxide particle nanocomposites, the synthesis or preparation procedures, and the most frequent applications of these materials.

Aside from metallic, metal oxide, or other inorganic nanoparticles, various complex hybrid nanostructures have also been formed on the cellulose surface. The specific fibrous structure and high number of hydrogen bonds give the cellulose sufficient porosity and high specific surface area, which can serve as a matrix to support other functionalized materials and as a template to form various inorganic hierarchical nanostructures. New materials with interesting combinations of properties and functions have been prepared by combining different inorganic nanostructures and polymer chains with various cellulose structures. Other than cellulose supported or templated inorganic compounds, more complex hybrid modifications or decorations of the cellulose structures have also been studied and reported. Complex hybrid cellulose materials have a wide variety of potential applications (e.g., in medicine, pharmaceutical technology, chemical catalysis, electronics, and energy conversion [1,3,338,339,340]). The development of complex cellulose-based structures aims at the formation of hierarchical nanoparticle structures with specific tailored combinations of properties for various applications such as the optimization of the catalytic activity through a combination of two catalytic materials or the optimization of the antibacterial activity of wound dressings through a combination of two antibacterial agents. In addition, combinations of three or even more components with cellulose have been reported. For example, MnO_2_, polypyrrole, and cellulose nanocrystals were applied to form lightweight, highly porous, and flexible hybrid (inorganic–organic) materials based on the cellulose aerogel for super capacitive application. Supercapacitors are a highly propulsive area of research and have already found commercial application [1]. A complex combination of cellulose nanocrystals, Fe_3_O_4_, cationic polymer, SiO_2_, poly(vinylpyrrolidone), and cyclodextrine was studied and its potential application in the separation of pharmaceutical compounds was demonstrated. Similarly, the CNC/Fe_3_O_4_/Au (CNFeAu) nanocomposite was used as a support matrix for the immobilization and separation of papain from the reaction solution with a high enzyme loading; the conjugated biomolecules retained their high enzyme activity and exhibited high stability and reusability. Inorganic nanostructures can be synthesized in situ on the surface of the cellulose fibrillar structure or they can be admixed to the cellulose. In this way, ZnO nanoparticles were formed in the presence of various saccharides, and such saccharide capped ZnO nanostructures were further deposited on the pine cellulose fibers that were subsequently incorporated into the alginic acid sheets, producing paper materials with a high degree of immobilization of biomolecules for the detection of blood type. Instead of chemical methods, physical methods of nanostructure formation can also be applied. For example, cellulose–Cu and Al_2_O_3_ sandwich nanostructures were prepared by magnetron sputtering and RF reactive sputtering, producing promising materials for EMI shielding applications. Recently, cellulose paper substrates have attracted tremendous interest from both academia and industry as a platform for biosensing applications. Cellulose fibers have an interesting combination of features that makes this substrate advantageous or even ideal for conducting chemical and biological analyses comprising the passive transport of fluids through their capillary wicking, excellent biocompatibility and biodegradability, large surface-to-volume ratio, porous structure, high abundance, and most importantly, their low-cost [339,341]. Since most of these hybrid structures contain biological molecules (proteins or DNA), doubt about the stability of the reported sensing devices over time has risen because in most cases, it was not studied. In Scheme 7, the versatility of cellulose substrates, the formation processes of hybrid cellulose/metallic or metal oxide particles, and the application possibilities of these complex hierarchical structures are presented.

### 6.1. Cellulose Supported Metallic Hybrid Nanostructures

**Figure 15:**
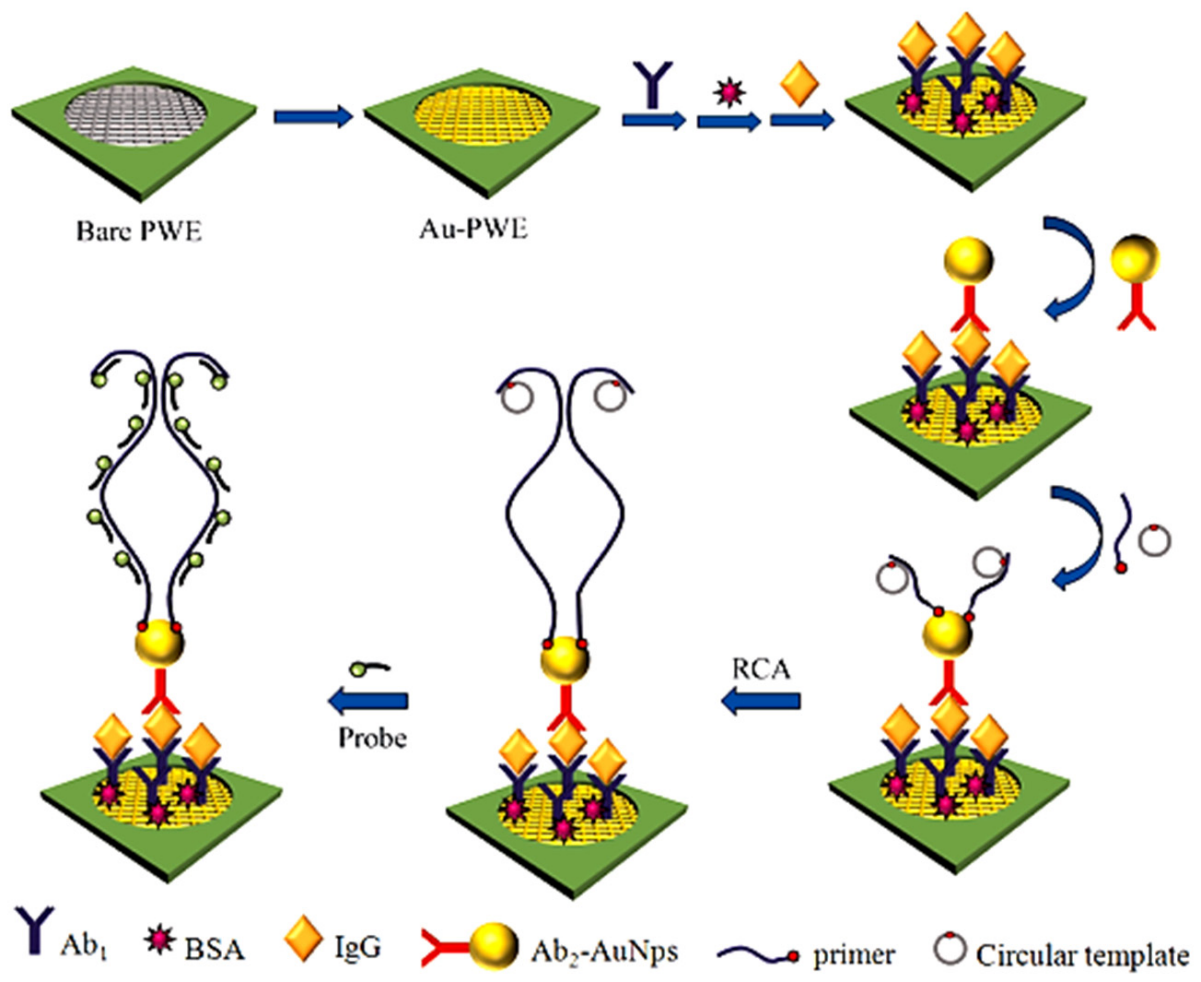
The schematic presentation of the fabrication procedures for the paper-based electrochemiluminescence origami device (PECLOD), (IgG—human immunoglobulin G protein; Ab—capture antibodies of IgG; BSA—bovine serum albumin; Ab_2_—monoclonal antihuman IgG antibodies; Au Nps—gold nanoparticles; primer—primer DNA; circular template—circular template DNA; probe —carbon dots-DNA probe; RCA—rolling circle amplification). Reprinted from [342]. Copyright 2015, with permission from Elsevier B.V.

A sensitive paper-based electrochemiluminescence origami device (PECLOD) for the detection of the human immunoglobulin G (H-IgG) antigen based on the rolling circle amplification (RCA) using the Au-paper working electrode (Au-PWE) was developed (Figure 15). The Au-PWE functionalized with the Ab1 antibodies provided a suitable environment for the efficient capture of H-IgG and played a significant role in electrochemiluminescence (ECL) amplification due to its 3D macroporous structure. Carbon dot (CD)-tagged RCA amplified DNA as a signal amplification device significantly increased the sensitivity and offered a high ratio of signal labels. The prepared PECLOD demonstrated the ultrahigh sensitivity, acceptable reproducibility, and broad dynamic range with very limited nonspecific adsorption under optimal conditions by applying H-IgG as a model protein. The designed PECLOD showed a dynamic response to H-IgG ranging from 1.0 fM to 25 pM with a very low detection limit of 0.15 fM. Additionally, the reported approach showed a potential to be extended for the detection of other biomarkers or proteins [342].

Ordinary filter paper with a 6 μm pore size was applied as a solid support that facilitated the impregnation with europium tetrakis dibenzoylmethide triethylammonium (EuD_4_TEA) and with gold nanoparticles that provided durability. This impregnated paper-based sensor platform was applied for the development of the fluorescence turn-on cyanide (CN) assay in aqueous media. The detection mechanism was based on two processes: (a) Ligand exchange of TEA with the CN^−^ ion stimulating the enhancement of cyanide specific fluorescence, and (b) the dissolution of gold nanoparticles, causing the recovery of fluorescence. The described CN assay demonstrated a visible transition of color as a function of CN^−^ exposure in the concentration range from 10^−2^ to 10^−12^ M. Additionally, an algorithm of image processing was developed that enabled the color change calibration and quantification of the CN^−^ concentration. The reported algorithm was applicable to an Android smart phone, thus transforming it into a quantitative detector of CN^−^ ions. The reported approach enabled sensitive, fast, and simple instrument free CN^−^ ion detection in water as well as the possibility of remote water monitoring and controlling in the laboratory [343].

By using cellulose nanocrystals (CNC) as a template, mesoporous silica (SiO_2_) films with chiral nematic ordering were prepared and further applied as a hard template for the formation of silver nanoparticles (AgNPs) from AgNO_3_ by reduction with NaBH_4_. The resulting SiO_2_ films deposited with the Ag NPs showed optical activities in the vicinity of the surface plasmon resonance (SPR) of the Ag NPs, as shown by the circular dichroism (CD) measurements. By conducting the experiments with three different SiO_2_ hard templates loaded with AgNPs dried and soaked with water, it was shown that the optical activities only originated from the long-range organization of the Ag NPs in the nematic chiral silica template. Since the SPR of metallic NPs is important in biosensing, the application of the response based on the chiral organization of metallic AgNPs embedded in a chiral mesoporous host will lead to the development of new biosensors. This is a showcase of the indirect application of CNC nematic liquid crystals in the formation of optically active inorganic structures [344].

**Figure 16:**
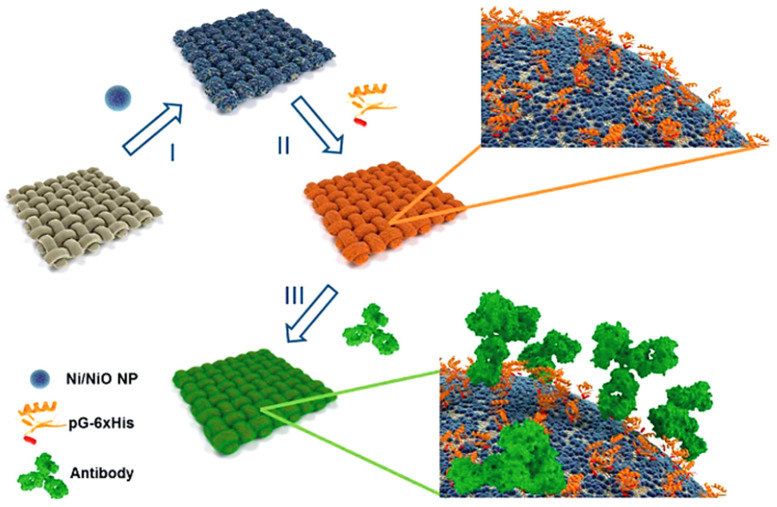
The schematic presentation of the bioactive scaffold preparation: (I) adsorption of Ni/NiO NPs on cellulose fiber-filter paper, (II) targeted immobilization of the polyhistidine-tagged protein G (pG-6xHis), and (III) protein G-mediated capture of antibodies. Protein G is presented by the protein G B1 domain (PDB: 1GB1, orange) bearing a 6xHis at its C-terminus (red). The antibody is an IgG2a immunoglobulin (PDB: 1IGT, green). Reprinted with permission from [345]. Copyright 2014 American Chemical Society.

Additionally, cellulose fibers (paper) have been used for the preparation of large surface area bioactive hybrid scaffolds by exploiting the self-assembly and adsorption of the previously formed oxidized nickel (Ni/NiO) nanoparticles (NPs). The Ni/NiO NPs enabled the oriented binding of functional His-tagged protein G to the cellulose surface as it was confirmed by the subsequent binding of fluorescent antibodies. It was shown that the interaction between the polyhistidine-tagged protein and the nickel substrate was chemically inert and highly resilient. The targeted conjugation of His-tagged protein G enabled the oriented binding of anti-Salmonella antibodies, enabling the attachment of Salmonella bacteria to the substrate (Figure 16). This simple approach opens the way for the application of other paper-supported metallic NPs as docking sites for a variety of biomolecules—nucleic acids or proteins. The reported strategy is based on the high surface area of the fibrillar cellulose structure, which enables a higher potential of protein capture, causing a large increase in the signal detection and improved assay sensitivity [345].

α-Cellulose was applied as a support in the formation of hybrid zinc oxide/silver (ZnO/Ag) nanostructures on its surface by the microwave solvothermal synthesis using Zn(OOCCH_3_)_2_ and AgNO_3_ as precursors and hexamethylenetetramine as the precipitating and reducing agent. Before synthesis, the surface of cellulose was activated by H_2_O_2_ with a relatively mild effect on the contact angle with H_2_O. The formed ZnO/Ag nanostructure was a nanodispersed semiconductor/metal hybrid with an exceptional collective plasmonic structure never previously observed. The experiments with a single precursor solution enabled the separate study of the interaction of Zn^2+^ and Ag^+^ ions with the cellulose surface and in this way, the reaction mechanism in the mixed precursor solution was explained. A specific interaction between the Zn^2+^ and the cellulose substrate was confirmed while no specific non-thermal effects of microwave heating were detected [346].

### 6.2. Cellulose Supported Metal Oxide Hybrid Nanostructures

For the antibody immobilization, pine cellulose fiber sheets decorated with saccharide capped-ZnO nanoparticles have been applied as a bioactive substrate. Initially, ZnO nanoparticles were formed in the presence of starch and alginic acid as well as sucrose and glucose. The fibers of pine cellulose were subsequently surface modified by the prepared saccharide capped ZnO nanoparticles that were finally incorporated into sheets of alginic acid. The immobilization of antibodies on the surface of the fibers was significantly enhanced by the adsorbed ZnO nanoparticles. The observed retention of antibodies was about 95% after rewetting the ZnO–alginic acid modified sheets with the saline solution. For the possible application of bioactive- or biosensing-papers for the detection of blood types, a high degree of the biomolecule immobilization is an important characteristic. The possibility of detecting blood types was successfully demonstrated by using A-, B-, and D-antibodies. Additionally, the ZnO nanoparticles introduced a strong activity against bacteria (*S. aureus* and *E. coli*) as well as strong resistance toward cellulase producing fungus *G. trabeum* [347].

Natural cellulose was applied to template the TiO_2_ and to prepare a bioinspired nanocomposite composed of nanotubular TiO_2_ and platinum quantum dots. TiO_2_ nanotubes were formed by calcination at 450 °C where the cellulose structure played the role of a sacrificial template. The studied composites were composed of three-dimensional hierarchical structures of TiO_2_ and metallic ultrafine Pt quantum dots with sizes of app. 2 nm being uniformly formed on the surface of the TiO_2_ nanotubes by a photo deposition process. Complex nanocomposite structures with 1.06 wt.% of Pt content demonstrated the maximum photocatalytic hydrogen production of 16.44 mmol h^−1^ g^−1^ from water-splitting, while higher concentrations of Pt caused a decrease in the photocatalytic activity. The sufficient photocatalytic stability was a result of the structural integrity of the nanocomposite during cyclic water-splitting processes. The introduction of cellulose supported hierarchical nanomaterials into photocatalytic systems is an innovative approach to the design and production of new photocatalytic systems [348].

ZnO nanorod arrays (NRAs) were formed on the CuO nanospindle (CuO NS)/paper by using a hydrothermal process to prepare the cellulose supported p–n junction device. Soaking with an aqueous solution of Cu(NO_3_)_2_ and hexamethylenetetramine and thermal treatment at 75 °C for 6 h produced cellulose fibers decorated with CuO NSs. The resulting CuO NSs/paper was coated with ZnO seeds and subsequently transformed into ZnO NRAs through hydrothermal synthesis at 85–90 °C for 3 h. The prepared device exhibited the I–V characteristics with rectifying behavior at 0.93 V of the turn-on voltage. This simple process for the ZnO NRA formation on the CuO NS-modified paper proved to be a useful strategy toward the formation of paper-based nanostructures for applications in electronic devices [349].

**Figure 17:**
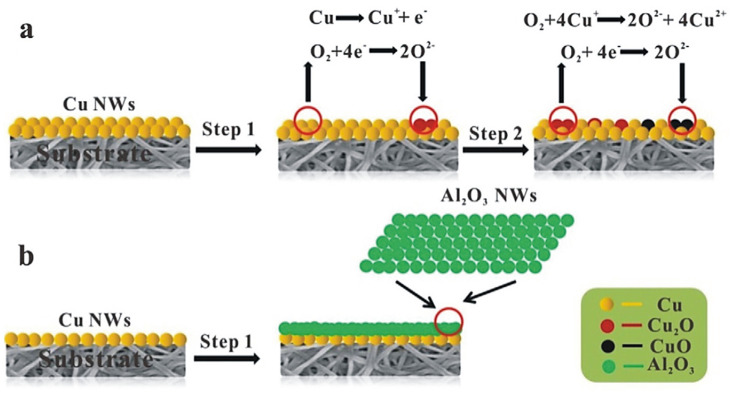
The schematic diagram of the anti-oxidation mechanism of the Cu nanostructure layers (**a**) in the absence and (**b**) presence of Al_2_O_3_ layers. Reprinted from [350]. Copyright 2016, with permission from Elsevier Ltd.

EMI shielding-based sandwich nanomaterials were prepared by functionalizing the bacterial cellulose with Cu and Al_2_O_3_ nanoparticles by using the magnetron sputtering and RF reactive sputtering method. To optimize the material properties, the ratio of the Cu and Al_2_O_3_ nanostructure films was explored by applying the P–B ratio theory. The addition of Al_2_O_3_ nanosheets enhanced the EMI shielding and anti-oxidation properties of the Al_2_O_3_/Cu/BC nanocomposite materials (Figure 17). The as-prepared sandwich nanomaterials thus demonstrated high EMI shielding properties (65.3 dB), high hydrophobicity, and good mechanical properties (41.3 MPa). The reported innovative topological composite cellulose structure proved to be a promising material for commercial applications in the area of EMI shielding [350].

Fe_3_O_4_ nanoparticles were formed in situ on the bacterial cellulose (BC) modified with fluoroalkyl silane (FAS) under moderate reaction conditions to prepare the magnetic flexible hybrid membranes with an amphiphobic surface. The surface chemical properties of the BC nanofibers (ether and hydroxyl groups) and the ultrafine network architecture both functioned as effective nanoreactor cavities for the formation of magnetite nanostructures. The FS10 sample with homogeneously dispersed magnetite nanoparticles on the BC showed the maximal amphiphobicity with an oil contact angle of 112° and a water contact angle of 130°. It also showed the excellent superparamagnetic properties (*M_s_* of 8.03 emu g^−1^) as well as the highest tensile strength (186 MPa) among the fluorinated magnetic membranes, and magnetic actuation was also demonstrated. This is an effective process in which to prepare self-cleaning, magnetic, and flexible BC membranes that can find potential application in EMI shielding, information storage, magnetographic printing, or as electronic actuators [351].

**Figure 18:**
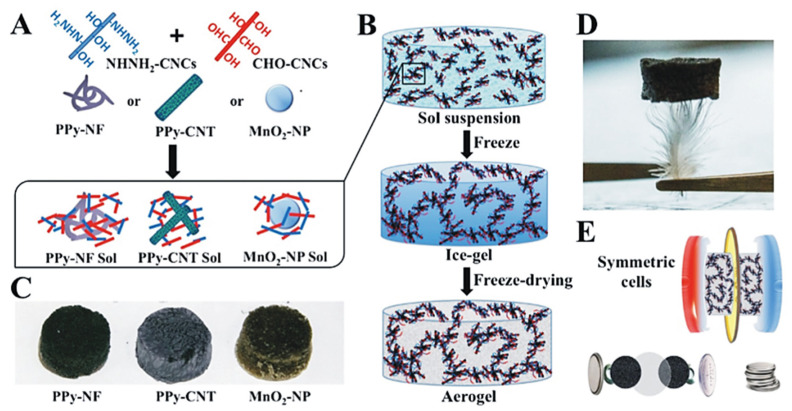
(**A**) The schematic presentation of the aerogel components including functionalized CNCs and capacitive nanoparticles, which form the initial suspension of cross-linked clusters. (**B**) The sol–gel process used to prepare aerogels. (**C**) Photograph of the final hybrid aerogels with a diameter of 1.5 cm. (**D**) Photograph emphasizing the lightweight nature of a hybrid aerogel resting on the top of a feather. (**E**) Schematic presentation showing the fabrication of symmetric supercapacitor cells in the CR2032-type coin-cell cases with two hybrid aerogels separated by a porous polyethylene membrane in a saturated Na_2_SO_4_ aqueous electrolyte [352]. Copyright 2015 John Wiley & Sons Inc. Reproduced with permission.

A simple aqueous sol–gel process was developed which comprised mixing, freezing, and freeze-drying to entangle one of the three types of capacitive nanoparticles with different dimensions, shapes, and chemistries within chemically modified CNC networks composed of aldehyde modified CNCs and hydrazine modified CNCs. Capacitive structures such as MnO_2_ nanoparticles, polypyrrole-nanofibers, and polypyrrole coated carbon nanotubes were synthesized separately and subsequently deposited into the 3D cellulose aerogel structure in one step during the aerogel assembly, providing a huge accessible surface area of the capacitive material, thus enhancing the charge storage (Figure 18). Because of the high mass ratio of the active material to the total electrode mass, excellent capacitance retention at high charge–discharge rates was observed. Multiple channels in the aerogels formed a higher number of electronic and ionic diffusion paths, producing a CNC-based material with the lowest internal resistance for nanocellulose supercapacitors. The preparation and supercapacitive performance of highly porous, lightweight, and flexible hybrid (inorganic–organic) materials based on CNC aerogels have been demonstrated [352]. 

**Figure 19:**
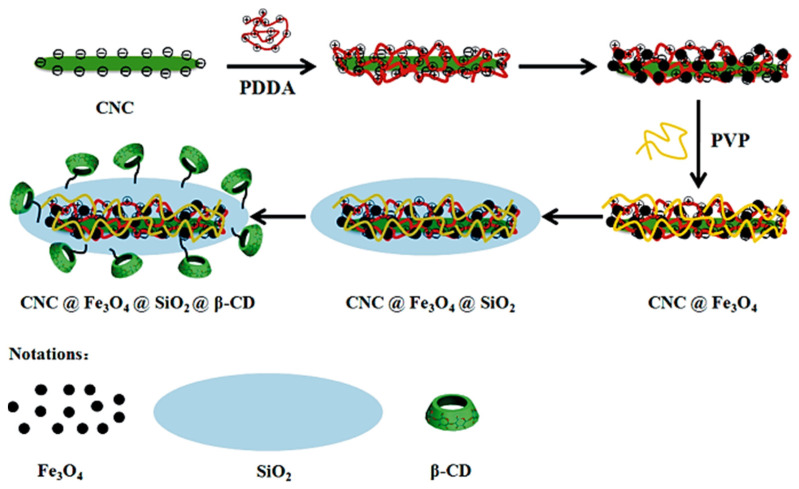
The schematic presentation for the synthesis of the CNC@Fe_3_O_4_@SiO_2_@ β-CD^a^. Reprinted with permission from [353]. Copyright 2014 American Chemical Society. ^a^CNC: cellulose nanocrystal. PDDA: poly(diallydimethylammonium chloride). PVP: polyvinylpyrrolidone. β-CD: β-cyclodextrin.

Some authors have combined cellulose structures, inorganic nanoparticles, and various polymers to obtain materials with highly complex structures and specific functions. For example, Chen et al. reported on a six-stage modification of CNCs [353]. The first stage was the surface deposition of poly(diallyldimethyl ammonium chloride) followed by the surface decoration with Fe_3_O_4_ nanoparticles (Figure 19). Subsequently, PVP was deposited on the surface of Fe_3_O_4_ decorated CNCs, followed by embedding the whole structure into SiO_2_ (Figure 19). The silica coating significantly increased the CNCs’ thermal stability since the onset decomposition temperature was shifted by 60 °C toward higher temperatures while Fe_3_O_4_ introduced magnetic properties into the system. On the surface of the final SiO_2_ layer, cyclodextrin molecules were finally grafted, which provided functionality to these complex structures (Figure 19). The as-prepared CNC/Fe_3_O_4_/SiO_2_/β-CD hybrids showed interesting magnetic properties and effective adsorption properties toward two pharmaceutical molecules as model compounds. Consequently, they were highly effective in the magnetic separation of imipramine hydrochloride and procaine hydrochloride from the water solution. The developed process has the potential to be used for the formation of other organic−inorganic nanocomposites by applying CNCs as a template and support.

**Figure 20:**
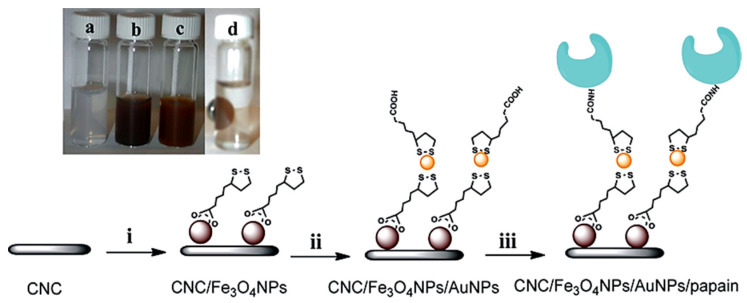
The schematic presentation of the stepwise formation of the conjugate of CNC/Fe_3_O_4_NPs/AuNPs/Papain—(**i**) FeCl_3_, FeCl_2_, thioctic acid; (**ii**) HAuCl_4_, α-cyclodextrin, NaBH_4_, thioctic acid; (**iii**) EDC, NHS, papain. Inset shows photographs of (**a**) pristine CNC; (**b**) CNC/Fe_3_O_4_NPs; (**c**) CNC/Fe_3_O_4_NPs/AuNPs; and (**d**) CNC/Fe_3_O_4_NPs/AuNPs attracted to the sidewall with a permanent magnet. Reprinted with permission from [354]. Copyright 2013 American Chemical Society.

The synthesis and characterization of Fe_3_O_4_ NPs and Au NPs immobilized on CNCs was reported. The CNC/Fe_3_O_4_NPs/AuNPs (CNFeAu) nanocomposite was developed as a support for the adsorption and separation of papain, having a maximum loading of 186 mg protein g^−1^ on the CNFeAu from the reaction solution (Figure 20). The conjugated papain molecules exhibited high stability and reusability and their high enzymatic activity was preserved. The enzyme adsorbed on CNFeAu retained more than 83% of the original activity, with high thermal tolerance and reusability compared to the nonadsorbed reference, as shown by the electrochemical detection and conventional spectrophotometric assay of the specific enzyme binding to the Thc-Fca-Gly-Gly-Tyr-Arg inhibitory film. The magnetic CNFeAu proved to be an effective support matrix with the potential for the magnetic separation of molecules as well as in immobilizing proteins, enzymes, and other biomolecules [354]. 

Mesoporous TiO_2_ containing a replica of a chiral nematic structure was synthesized by preparing a titania aqueous sol using a titanium isopropoxide precursor and acetic acid and subsequently mesoporous TiO_2_ with a chiral nematic structure (CNTiO_2_) was formed by dipping the previously prepared CNC photonic films, prepared by an evaporation-induced self-assembly (EISA) method, into the TiO_2_ sol. CNC photonic films acted as a chiral biotemplate, which was removed by calcination at 500 °C for 4 h and mesoporous chiral TiO_2_ film was formed. The stopband of the TiO_2_ photonic films was controlled by the quantity of D-glucose added to the system. Finally, mesoporous TiO_2_ was decorated with Au nanoparticles using HAuCl_4_ as a precursor and NaBH_4_ as a reducing agent to increase its photocatalytic activity. The prepared mesoporous chiral TiO_2_/Au hybrid films were tested as catalysts in the photocatalytic splitting of water for the production of H_2_. It was demonstrated that the photo generation of H_2_ was more than eight times higher compared to the conventional mesoporous TiO_2_ loaded with Au nanoparticles [355].

The number of publications on cellulose supported or templated hybrid inorganic nanostructures is extensive due to the large number of hybrid combinations and potential applications. These are summarized in Table 6 to concentrate the information in the smallest possible space. The majority of publications in Table 6 reported on the formation of cellulose fiber (paper)–Au or other noble metal based electrodes, constituting various medical and environmental sensors. Noble metals predominate due to their stability and resistance to corrosion. Usually, metallic nanoparticles are formed in two stage process: First, the formation of seeds or nuclei, followed by the seeded growth of nanostructures that completely cover the cellulose surface and form conductive electrodes. The most commonly used precursors are HAuCl_4_, H_2_PtCl_6_, AgNO_3_, or H_2_PdCl_4_ while the most frequently used reducing agents are NaBH_4_, Na citrate, or ascorbic acid. The most frequently used process for the growth of Au nanorods is the one reported by Busbee et al. [356]. The most important area of their application is in medical diagnostics and environmental monitoring. The most predominantly applied cellulose substrate is paper—cellulose fibers but bacterial nanocellulose (BNC) and cellulose nanocrystals (CNCs) are also used.

## 7. Discussion, Conclusions, and Perspectives

### 7.1. Discussion

By intensifying the global climate changes and shortages in natural resources, increased attention has been devoted in recent years to sustainable resources and environmentally-friendly solutions. Cellulose is the most abundant natural polymer on the Earth, with an annual production of ~1.5.10^12^ tons, which is comparable to the reserves of the main fossil and mineral resources on our planet. Cellulose, with its fibrillary structure, contributes greatly to the diversity in the field of the synthesis and application of hierarchical inorganic nanostructures with additional alternatives. Based on the large number of hydroxyl groups on its surface, it influences the formation of inorganic nanomaterials. It serves as a support or template, it can control crystallization or act as a nanoreactor for in situ precipitation, and it can also prevent their aggregation and agglomeration to a large extent. Cellulose in any form enables the formation of hierarchically organized organic/inorganic hybrid nanostructures that cannot be obtained otherwise.

There are two types of cellulose/inorganic composite structures: (i) Cellulose supported inorganic composite materials and (ii) pure inorganic materials with cellulose templated porosities. In the first type, cellulose is present in the final composite material while in the second case, it serves as a sacrificial template and is removed by calcination, leaving the pure inorganic material. The inorganic component can be synthesized or formed in situ on the surface of the cellulose or it can be synthesized ex situ, and subsequently deposited on the cellulose. In the first case, cellulose serves as a template and support, while in the second case, it only serves as a support. When the inorganic (nano)structures are formed or grown on the surface of the cellulose, its surface characteristics (surface morphology, concentration, and reactivity of hydroxyl groups and the space dimensions between the cellulose fibrils) affect and control their formation. On the other hand, when the cellulose is removed by calcination and a porous inorganic material is obtained, then the dimensions of the cellulose structures become important since they directly or indirectly define the dimensions of the pores in the inorganic matrix. The cellulose aerogel, as an example, acting as a support, produces lightweight, highly flexible, porous, mechanically resistant, and environmentally-friendly structures accompanied with interesting catalytic, electronic, optical, magnetic, and antibacterial properties. The potential areas of applications are very broad (i.e., antimicrobial agents in medicine, heterogeneous catalysis in chemical technology, energy conversion and storage, selective adsorption of metallic ions or organic compounds for water purification, as well as sensors in electronics and medical diagnostics).

The most widely studied area of application is medicine, where hydrophilic cellulose aerogels are combined with metallic Ag or/and ZnO nanoparticles to achieve the antibacterial effect. There are numerous publications reporting on the enhanced inhibiting effect on the bacterial or fungal growth. In many cases, the cellulose supported nanoparticles showed higher antibacterial activities compared to the neat nanoparticles, thus presenting a highly promising alternative for antimicrobial wound dressings or antimicrobial packaging materials. Highly promising biomaterials are cellulose/hydroxyapatite hybrid materials used as bone-tissue scaffolds for bone healing.

Heterogeneous catalysis is another application area of cellulose-templated inorganic nanostructures. Metallic or metal oxide nanoparticles immobilized on the cellulose structures show superior catalytic activities compared to conventional catalytic systems while the removal of a catalyst is not the issue, since nanoparticles are attached to cellulose structures. Cellulose templated pure inorganic materials also showed enhanced activities in heterogeneous catalysis due to their porosity and high specific surface area. The highly important growing area comprises catalytic systems in alternative energy sources such as fuel cells and high capacity batteries.

Supercapacitors based on nanocellulose supports with high charge storage, excellent capacitance retention, and very low internal resistance represent another fast growing application field of cellulose supported inorganic or hybrid nanostructures.

The selective and efficient adsorption of toxic metallic ions such as As^5+^, Pb^2+^, Cd^2+^, Cr^6+^, Mn^2+^, etc. from the aqueous medium gives the cellulose supported inorganic structures a high potential for use as a water decontaminant, and also in the purification of water or air from various organic chemicals. 

Another highly promising area of application includes fast response humidity, chemical, and biomedical sensors, where the porous structure of cellulose-based paper with a high specific surface area certainly has advantages over conventional materials. The rapidly growing subfield of microfluidic cellulose fiber (paper)-based analytical devices is a highly promising approach toward simple, portable, disposable, and low-cost devices for molecular analysis, environmental detection, and health monitoring.

### 7.2. Conclusions

A comprehensive review on the application of cellulose in all of its forms as a support or template for various inorganic materials demonstrates the role of cellulose as a highly versatile support and template for the formation of inorganic nanostructures as well as for bioinspired hierarchical structures and assemblies with interesting combinations of properties. Cellulose, as a fibrillar and functional biomaterial support or template, offers new approaches and pathways to the nucleation and controlled growth of inorganic nanoparticles with a defined shape and porosity as well as to hierarchically organized nanostructures that are difficult or even impossible to prepare through conventional processes. Cellulose, and especially cellulose nanostructures with abundant hydroxyl functionalities on their surface, can intensively interact with various metallic ions. 

Cellulose thus influences the formation mechanism of metallic and metal oxide nanoparticles and serves as a templating agent, which enables the preparation of (nano)structures that cannot be obtained by conventional methods. Due to the strong interactions of hydroxyl groups with inorganic ions, cellulose prevents the aggregation and agglomeration of growing inorganic nanostructures, indicating a higher interaction energy of these structures with the cellulose surface compared to the driving energy of particle aggregation and Ostvald ripening. Since the cellulose-supported nanostructures do not agglomerate and grow into larger structures, they retain their nanodimensional size and extremely large specific area, which is highly important in the heterogeneous catalysis of various chemical reactions. 

However, the formation and growth of inorganic nanostructures should be performed in a more sustainable way to reduce the use of toxic compounds and waste generation as well as by applying low temperature aqueous solution synthetic protocols, thereby reducing the production costs and enhancing manufacturing safety. Additionally, the implementation of such structures may cause some environmental issues associated with their application. The environmental impact should be assessed from many different aspects (waste, biomagnification in the food chain, transport, etc.) including their potential toxicity when released into ecosystems or the human body. The application of cellulose in any form offers answers to the majority of the previously mentioned issues. 

The review of publications up to the present shows that research in this area is heading away from the simple deposition of one type of inorganic nanostructure onto the (nano)cellulose structure toward the deposition of hybrid structures combining metallic, metal oxide, or sulfide with carbon nanotubes or graphene oxide or conducting polymers. These cellulose-supported hybrid inorganic materials form functional hierarchical (nano)structures with targeted applications in (photo)catalysis, Li-batteries, bone tissue engineering, supercapacitors, wound healing, waste water cleaning, colorimetric analysis systems, in sensing, or in other areas of electronics. 

Recent advances in the preparation of cellulose-based inorganic nanostructures suggest that the cellulose application will facilitate overcoming the major drawbacks of traditional synthetic methodologies. This review provides an insight into a rather specific area of cellulose-based inorganic (nano)structures and the trends of their exploitation in specific applications, demonstrating their huge potential to become as important as other well-known cellulose applications such as a bioreplacement for fossil fuels, organic chemicals, and/or organic materials based on fossil fuels. A large number (~500) of publications on this topic in the last ten years, mostly in the high impact scientific literature, strongly supports the growing importance of cellulose-based inorganic (nano)structures in materials, technologies, and applications of the future.

### 7.3. Perspectives

Biomaterials based on cellulose are emerging as highly promising solutions to a number of technological challenges. Cellulose and cellulose-based materials are abundant and biocompatible but also possess structures for transformative device performance. A special case is that of cellulose–inorganic hierarchical nanostructures, since this scientific field is a combination of nanotechnology and environmental science. Currently, a substantial number of promising devices and applications of hybrid cellulose–inorganic structures have been demonstrated, but there are still important challenges in the basic scientific research and understanding to bring cellulose-based hybrid materials to a commercial reality in many innovative applications such as: energy saving and transformation applications, sustainable catalytical systems, green electronics, innovative low-cost sensors or detectors, and biological or biocompatible devices. These challenges comprise (i) low-cost solutions in the extraction of cellulose structures from wood or other cellulose resources and in the production of either components or devices; (ii) high performance cellulose–inorganic hierarchical nanomaterials and devices based on these materials with suitable lifetime and durability; and (iii) the integration of systems composed of multiple devices.

Innovative developments using cellulose–inorganic hybrid materials elaborated in this review, especially those based on nanocellulose, will generate new solutions influencing our everyday life in the future. To cope with the emerging global challenges, environmentally-friendly and forward-thinking strategies are expected to sustain the research initiatives, and cellulose and cellulose–inorganic hierarchical materials will certainly play an important and decisive role in this demanding task.

## Figures and Tables

**Table 1:** The list of publications on cellulose supported metallic nanostructures. The list is organized from large cellulose structures (CF and RC) via microstructured celluloses (MCC and MFC) to nanostructured celluloses (BNC and CNCs).

Inorg. Particle (Size—nm)	Cellulose Used	Precursor (Synthetic Path)	Application	Reference
**Precious Metallic Cellulose Supported Nanostructures**				
Au NPs (~2 nm)	CF	Bis(ethylenediamine) Au(III)Cl_3_, (CH_3_)_2_Au(III) acetylacet.,(red.by NaBH_4_)	Catalyst (glucose oxidation)	[88]
Ag NPs (8–20 nm)	RC	AgNO_3_ (hydrothermal reduction)	Catalyst (reduction of 4-nitrophenol)	[89]
Au NPs (<5 nm)	MFC	HAuCl_4_ (reduction by NaBH_4_)	Catalyst (reduction of 4-nitrophenol)	[90]
Au–Ag NPs	MFC		Conductive nanofiller	[91]
Au–Pd NPs (4–9 nm)	MFC	HAuCl_4_, [Pd(NH_3_)_4_]·Cl_2_ (reduction by NaBH_4_)	Catalyst (reduction of 4-nitrophenol)	[92]
Au/Ag NPs	BNC	HAuCl_4_, AgNO_3_ (reduc. by poly(ethyleneimine)	-	[93]
Au NPs	BNC	HAuCl_4_ (reduction by cellulose)	Conductive nanofiller	[94]
AuNR@AgNCs	BNC	HAuCl_4_, NaBH_4_, AgNO_3_, CTAC	SERS–detection of TNT	[95]
Au NPs (2–10 nm)	CNCs	HAuCl_4_, trisodium citrate, oleylamine, mercaptocation	Chiral photonic materials	[96]
Au NPs (20–30 nm)	CNCs	HAuCl_4_, NaOH (reduction by CNCs)	Photothermal nanocomposite materials	[97]
Au NPs (30.5 nm)	CNCs	HAuCl_4_ (reduction by cellulose)	Catalyst (reduction of 4-nitrophenol)	[98]
Au NPs (4.5–7.1 nm)	CNCs	HAuCl_4_ (reduction by NaBH_4_)	Biosensor (for 2-mercaptoethanol)	[99]
Au NPs (2–3 nm)	CNCs	HAuCl_4_ (reduction by—HS groups on the CNC surface)	Catalyst (alkyne–aldehyde–amine-coupling)	[100]
Au NPs (2–4 nm)	CNCs	HAuCl_4_ (reduced with no and by NaBH_4_)	Catalyst (reduction of 4-nitrophenol)	[101]
Au NPs (~3 nm)	CNCs	HAuCl_4_ (reduction by NaBH_4_)	Catalyst (reduction of 4-nitrophenol)	[102]
Au, Ag NPs	CNCs	HAuCl_4_, AgNO_3_, ascorbic acid	Catalyst (reduction of 4-nitrophenol, 4-aminophenol)	[103]
Au NPs(30–80 nm)	CNF	HAuCl_4_, Na_3_Cit·2H_2_O	Substrate–SERS spectroscopy	[104]
Au NPs (30–80 nm)	CNCs	HAuCl_4_, K_2_CO_3_, NaOH	Seeds for Au coating of CNCs with tunable optical properties	[105]
Au NPs (35 nm)	CNCs	HAuCl_4_ (reduction by trisodium citrate)	Sorption and detection of Au nanoparticles in H_2_O	[106]
Pt NPs (1–5 nm)	CF	H_2_PtCl_6_, Na rhodizonate	Catalyst (reduction of 4-nitrophenol, methyl orange)	[107]
Pt NPs	CNF	H_2_PtCl_6_, (reduction by NaBH_4_)	Catalyst (reduction of 4-nitrophenol)	[108]
Pt NPs (5–30 nm)	CNCs	H_2_PtCl_6_ (reduction by CNCs)	—	[109]
Pt NPs (11–101 nm)	CNCs	H_2_PtCl_6_ (reduction by wood nanomaterial)	Catalyst (reduction of 4-nitrophenol)	[110]
Pt NPs (~2 nm);	CNCs	H_2_PtCl_6_ (reduction by CNCs)	Electrocatalyst (oxygen reduction)	[111]
Pd NPs (~20 nm)	BNC	PdCl_2_ (reduction by KBH_4_)	Catalyst (Heck reaction)	[112]
Pd NPs (~20 nm)	BNC	K_2_PdCl_4_ (reduction by NaBH_4_)	Catalyst (Suzuki–Miyaura reaction)	[113]
Pd NPs (3.6 nm)	CNCs	PdCl_2_ (reduction by H_2_)	Catalyst (hydrogenation of phenol; Heck Coupling)	[114]
Pd NPs (1–7 nm)	CNCs	PdCl_2_ (reduction by CNCs)	Catalyst (red. of methylene blue and 4-nitrophenol)	[115]
Pd-Cu NPs	BNC	PdCl_2_ and CuCl_2_ (reduction by KBH_4_)	Catalyst (water denitrification)	[116]
Ru NPs (~8 nm)	MFC	RuCl_3_ (reduction by NaBH_4_)	Catalyst (aerobic oxidation of benzyl alcohol)	[117]
Ag-Au NPs (8–10 nm)	CF	AgNO_3_, HAuCl_4_, NaOH, urea	Antibacterial agent	[118]
Ag NPs	CF	RF sputtering	Antibacterial agent	[119]
Ag NPs (3–15 nm)	MCC	AgNO_3_, UV light reduction	Catalyst (reduction of p-nitrophen. to p-aminophen.)	[120]
Ag NPs (~6 nm)	MFC	AgNO_3_ (reduction by NaBH_4_)	Catalyst (reduction of rhodamine B)	[121]
Ag nanowires	MFC	Previously formed Ag nanowires	Conductor in transparent nanopaper	[122]
Ag NPs (3–4 nm)	MFC	AgNO_3_, UV light triggered reduction by MFC	Aerogels	[123]
Ag nanowires	MFC	Previously formed Ag nanowires	Conductive nanofiller	[124]
Ag NPs (~4 nm)	MFC	AgNO_3_ (reduction by NaBH_4_)	Catalyst (aza–Michael reaction)	[117]
Ag NPs (~6 nm)	MFC	AgNO_3_ (reduction by NaBH_4_)	Antibacterial agent	[125]
Ag nanowires	MFC	-	Conductive nanofiller	[126]
Ag NPs	BNC	AgNO_3_ (reduction by sodium citrate)	SERS substrate-pesticides detection	[127]
Ag NPs (var. sizes)	CF, MCC, CNCs	AgNO_3_, (reduction by cellulose)	-	[128]
Ag NPs (<10 nm)	BNC	AgNO_3_ (reduction by NaBH_4_)	Antibacterial agent	[129]
Ag NPs (17 nm)	BNC	AgNO_3_ (reduction by cellulose)	Antibacterial agent	[130]
Ag NPs (~30 nm)	BNC	AgNO_3_ (red. by NH_2_NH_2_, NH_2_OH, ascorbic acid)	Antibacterial agent	[131]
Ag NPs (8–15 nm)	BNC	AgNO_3_ (reduced by triethanolamine)	Antibacterial agent	[132]
Ag NPs (~16 nm)	BNC	AgNO_3_, (reduction by BNC)	Antibacterial agent	[133]
Ag NPs (5– 50 nm)	CNFs	AgNO_3_ (reduction by Na citrate)	Flusilazole adsorption and analysis	[134]
Ag NPs	CNFs	AgNO_3_ (reduction by Na citrate)	SERS probe for carbendiazim	[135]
Ag NPs (10–50 nm)	CNCs	AgNO_3_ (reduction by CNC)	Electrocatalyst (reduction of oxygen)	[136]
Ag NPs (<10 nm)	CNCs	AgNO_3_ (reduction by NaBH_4_)	DNA biosensor	[137]
Ag NPs (10–15 nm)	CNCs	AgNO_3_ (reduction by NaBH_4_)	Antibacterial agent	[138]
Ag NPs (1 nm–10 μm)	CNCs	AgNO_3_ (reduction by CNCs)	Antibacterial agent	[139]
Ag NPs (20–45 nm)	CNCs	AgNO_3_ (reduction by CNCs)	Antibacterial agent	[140]
Ag NPs (2–3 nm)	CNCs	AgNO_3_ (reduction by NaBH_4_)	—	[141]
Ag NPs (~10 nm)	CNCs	AgNO_3_ (reduction by dopamine)	Catalyst (reduction of 4-nitrophenol)	[142]
Ag NPs (~7 nm)	CNCs	AgNO_3_ (reduction with dopamine hydrochloride)	Antibacterial agent	[143]
Ag NPs (10–80 nm)	CNCs	AgNO_3_ (reduction by NaBH_4_)	—	[144]
Ag NPs (1–2 nm)	CNCs	Ag wire and AgNO_3_ (reduction by CNCs)	Catalyst (hydrogenation of aldehydes, nitrophenol, alkenes and alkynes)	[145]
Ag NPs (10–50 nm)	CNCs	AgNO_3_ (reduction by NaBH_4_)	Plasmonic activators for shape memory polymers	[146]
**Semi- and non-precious cellulose supported metallic nanostructures**				
Cu NPs	CF	CuSO_4_, poly(ethylenimine)	-	[147]
Cu NPs (~5 nm)	MFC	CuCl_2_ (reduction by ascorbic acid)	Catalyst for the reduction of 4-nitrophenol	[148]
Cu NPs	CNCs	Cu(OCOCH_3_)_2_, NaOH (reduction by ascorb. acid)	Conductive nanofiller	[149]
Cu NPs (50 nm)	CNCs	CuSO_4_, (reduction by ascorbic acid and NaBH_4_)	Catalyst for C–N coupling reactions	[150]
Cu NPs (10–20 nm)	CNCs	CuSO_4_ (reduction by hydrazine)	Catalyst for oxidation of sulfides and alcohols	[151]
Fe NPs (200–300 nm)	CF	FeSO_4_ (reduction by NaBH_4_)	Adsorbent for Cd(II) ions	[152]

**Table 2:** The adsorption properties of Cel–SH for the copper-catalyzed (cellulose supported Cu_2_O) Huisgen 1,3-dipolar cycloaddition of azide with the terminal alkyne forming 1-(benzyl-1H-1,2,3-triazol-4-yl)methanol [171]. Copyright 2016 John Wiley & Sons Inc. Reproduced with permission.

Entry ^[a]^	SH Loading	Yield ^[b]^	Cu Adsorbed
	(mol%)	[%]	[%]
1	0	<5	0
2	3.2	41	4
3	8	43	58
4	16	87	94
5	24	88	97
6	32	91	97.5

^[a]^ Reaction conditions: benzyl azide (1 mmol), propargyl alcohol (1.5 mmol), CuSO_4_·5H_2_O and Cell–SH were stirred in 5 mL of t-BuOH/H_2_O (1:1) for 14 h at the specified temperature. ^[b]^ Yield of the isolated product.

**Table 3:** The list of publications on the cellulose supported metal oxide nanostructures. The list is organized from large cellulose structures (CF and RC) via microstructured celluloses (MCC and MFC) to nanostructured celluloses (BNC and CNCs).

Inorg. Particle (Size—nm)	Cellulose Used	Precursor (Synthetic Path)	Application	Reference
**Cellulose Supported TiO_2_ Nanostructures**				
TiO_2_ NPs (5–100 nm)	CF	Ti(OBu)_4_, HCl (hydrothermal reaction)	Photocatalyst for CO_2_ reduction	[176]
TiO_2_ NPs (40–250 nm)	CF	Commercial TiO_2_ (hydrothermal treatment)	Photocatalyst, antibacterial agent	[177]
TiO_2_ NPs (~4 nm)	CF	TiCl_4_ (hydrothermal reaction—ultrasonication)	Photocatalyst in self-cleaning fabric	[178]
TiO_2_ NPs (10–20 nm)	CF	Ti(IV) isopropoxide (hydrothermal reaction)	Photocatalyst and antibacterial agent	[179]
TiO_2_ NPs	CF	TiOSO_4_, urea	Adsorbent for phosphate removal	[180]
TiO_2_ NPs	MCC	TiCl_4_, EtOH (sol–gel process)	Adsorbent for Pb^2+^, Cd^2+^, Zn^2+^ ions	[181]
TiO_2_ NPs (300–900 nm)	MCC	TiCl_3_, NH_3_ (hydrothermal reaction)	Photocatalyst (H_2_ production)	[182]
TiO_2_ nanorods (l.: 26 nm; w.: 16 nm)	MFC	Commercial TiO_2_ NPs	—	[183]
TiO_2_ NPs	MFC	Commercial TiO_2_ NPs	Antibacterial agent	[184]
TiO_2_ NPs-anatase	BNC	TiO_2_ powder, laser vaporization	Photocatalyst, purification of drinking water	[185]
TiO_2_ NPs (5–15 nm)	BNC	Ti(IV) n-butoxide, sol–gel process	Detector for phosphopeptides	[186]
TiO_2_ NPs	BNC	Ti(IV) n-butoxide Solvothermal process	Photocatalyst (methyl orange degradation)	[187]
TiO_2_ NPs	CNF	Commercial TiO_2_	Photoanodes	[188]
TiO_2_ NPs	BNC	Ti(IV) isopropoxide, Sol-Gel process	Antibacterial agent	[189]
TiO_2_ NPs	BNC	Commercial TiO_2_	Adsorbent for water contaminants	[190]
Black TiO_2_ NPs	BNC	Ti[OCH(CH_3_)_2_]_4_, microwave-assist. sonochem. process	Interfacial solar evaporator	[191]
TiO_2_ NPs	CNCs	TiOSO_4_ (hydrolysis with sulfuric acid)	UV absorber in skin care products	[192]
TiO_2_ NPs (50–100 nm)	CNCs	Commercial nano TiO_2_	Drug release system	[193]
**Cellulose supported iron oxide nanostructures**				
Fe_3_O_4_ NPs	CF	FeSO_4_, NH_3_, H_2_O_2_, PEG, previously formed Fe_3_O_4_	Magnetic fibers	[194]
Fe_2_O_3_ NPs (50–200 nm)	CF	FeSO_4_, NaOH (ultrasonication)	Magnetic paper	[195]
Fe_3_O_4_ NPs	CF	FeCl_3_, FeCl_2_, NaOH	Cancer treatment with 5-Fluorouracil	[196]
Fe_2_O_3_ NPs	CF	(NH_4_)_2_Fe(SO_4_)_2_, NaH_2_PO_2_,	Encapsulation and delivery-biologically active compounds	[197]
Fe_2_O_3_ nanoplatelet (48 nm)	RC	FeCl_2_, FeCl_3_ (reaction with NaOH)	—	[198]
Fe_3_O_4_ NPs	CF	FeCl_2_, FeCl_3_ (reaction with NaOH)	Immobilization of prenyltransferase NovQ	[199]
Fe_3_O_4_ NPs	MCC	FeSO_4_, FeCl_3_, NH_3_	Biotechnology, water purification	[200]
Fe_3_O_4_ NPs (~3 nm)	MCC	FeCl_3_, FeCl_2_, NH_3_	Antibacterial and contrasting agent	[201]
Fe_3_O_4_ NPs (~9 nm)	BNC	Fe(III) acetylacetonate, (microwave sol–gel process)	Magnetic paper	[202]
Fe_3_O_4_ NPs	BNC	Fe_2_(SO_4_)_3_, FeSO_4_, reaction with NaOH	Absorbing agent (removal of Cr(VI) ions)	[203]
Fe_3_O_4_ NPs	CNFs	Fe(NO_3_)_3_, FeSO_4_, NaOH, citric acid, nano TiO_2_	Recoverable catalyst-H_2_ photogeneration	[204]
Fe_3_O_4_ NPs (~10 nm)	CNCs	FeCl_2_, FeCl_3_, NH_3_ (precipitation)	Substrate for protein separation	[205]
Fe_3_O_4_ NPs	CNCs	FeCl_2_ and FeCl_3_ reaction with ammonia	Targeted delivery of doxorubicin	[206]
Fe_3_O_4_ NPs	CNCs	FeCl_2_, FeCl_3_ (reaction with ammonia)	Semiconductor in antistatic paper	[207]
**Cellulose supported ZnO nanostructures**				
ZnO nanorods	CF	(Zn(CH3COO)_2_, hexamethylenetetramine	Antibacterial agent in packaging	[208]
ZnO nanowires	CF	Previously synthesized ZnO NWs, Zn(CH_3_COO)_2_ (solvothermal hydrolysis)	Piezoelectric accelerometer	[209]
ZnO NPs (3–30 nm)	CF	Zn(CH_3_COO)_2_, reaction with NaOH	Antibacterial agent	[210]
ZnO nanorods	CF	Zn(NO_3_)_2_ (hydrothermal growth)	Semi-conductor	[211]
ZnO rods	CF	Zn(NO_3_)_2_, (CH_2_)_6_N_4_, (hydrothermal synthesis)	Antibacterial rubber	[212]
ZnO NPs	CF	Zn(NO_3_)_2_, urea (hydrothermal process)	Photocatalyst (methyl orange degradation)	[213]
ZnO nanorods	BNC	Zn(NO_3_)_2_, (CH_2_)_6_N_4_, (hydrothermal synthesis)	—	[214]
ZnO NPs	BNC	Zn(NO_3_)_2_, solution plasma process	Antibacterial agent	[215]
ZnO NPs (100–200 nm)	BNC	Zn(OOCCH_3_)_2_, solvothermal process	Antibacterial agent	[216]
ZnO NPs	BNC	Zn(NO_3_)_2_, NaOH	Antibacterial dressing for burn wounds	[217]
ZnO NPs	CNCs	Commercial nano ZnO	Semiconductor in flexible electronic applications	[218]
ZnO nanorod clusters	CNCs	Zn(CH_3_COO)_2_, reaction with NaOH	Antibacterial agent	[219]
ZnO NPs (10–50 nm)	CNCs	ZnCl_2_, NaOH	Catalyst in degrad. of tetracycline	[220]
**Cellulose supported miscellaneous oxide nanostructures**				
BiVO_4_	CF	Bi(NO_3_)_3_·5H_2_O, citric acid	Photocatalyst for methyl orange	[221]
Ti_3_(PO_4_)_4_	CF	Waste Ti metal, H_2_SO_4_, Na_3_PO_4_	Photocatalyst for crystal and methyl violet	[222]
MnO_2_ (150 nm)	MCC	MnSO_4_, KMnO_4_	Adsorbent of Pb^2+^, H_2_O purification	[223]
In_2_O_3_–10 wt.% SnO_2_	MFC	RF sputtering	Semiconductive nanofiller	[128]
Ag_2_O NPs (2–20 nm)	MFC	AgNO_3_, NH_3_, NaOH	Adsorbent for I^ˉ^ ions	[224]
H_3_PW_12_O_40_	BNC	Commercial PTA	Photochromic agent	[225]
Mn_3_O_4_ (300 nm)	BNC	KMnO_4_, reduction by carboxymethyl cellulose	Electrode material	[226]
ZnO-CdS NPs	BNC	Zn(NO_3_)_2_, CdSO_4_	Photocatalyst (degr. of methyl orange)	[227]
BaTiO_3_	BNC	Ti(OC_4_H_9_-n)_4_, H_4_Ba_6_(O)(OCH_2_CH_2_OCH_3_)_14_, hydrolysis	Piezoelectric paper	[228]
BaTiO_3_	CNCs	Commercial BaTiO_3_	Piezoelectric energy harvester	[229]
La_2_CuO_4_	BNC	La(NO_3_)_3_/Cu(NO_3_)_2_ hydrothermal process	Catalyst-methanol steam reforming	[230]
CoFe_2_O_4_	BNC	FeCl_3_ and CoCl_2_, reaction with NaOH	Magnetic paper	[231]
V_2_O_5_	BNC	Vanadium(V) oxytriisopropoxide hydrolysis with HCl	Semiconductive nanofiller	[232]
CuO NPs	BNC	Previously prepared CuO NPs	Antimicrobial agent	[233]
CuO NPs	BNC	Cu(OOCCH_3_)_2_, CH_3_COOH, ethylene glycol	Antibacterial agent	[234]
CuO NPs (~7 nm)	CNCs	CuSO_4_ (reduction by NaBH_4_)	Catalyst (reduction of 4-nitrophenol)	[235]
FeMnO_x_ (50–100 nm)	CF	KMnO_4_, FeSO_4_, NaOH, cetyltrimethyl ammonium Br	Adsorbent for As(III) and As(V) removal	[236]
CeO_2_ NPs (10–40 nm)	RC	Ce(NO_3_)_3_, NaOH	UV shielding	[237]

**Table 4:** The list of publications on cellulose-templated porous metal oxide structures or metal oxide nanostructures supported by carbon fibers formed from cellulose structures. The list is organized from large cellulose structures (CF and RC) via the microstructured celluloses (MCC and MFC) to the nanostructured celluloses (BNC and CNCs).

Inorg. Particle (Size—nm)	Cellulose Used	Precursor (Synthetic Path)	Application	Reference
**Cellulose Templated Inorganic Oxides**				
TiO_2_	MCC	titanium isopropoxide, HNO_3_, H_3_PO_4_, NaOH, urea	Sorbent microextr. of organic compounds	[262]
TiO_2_ nanofibers	CNF	titanium isopropoxide, NH_3_	Photocatalyst—degr. of methylene blue	[263]
TiO_2_ network	CNCs	Ti(N(CH_3_)_2_)_4_, atomic layer deposition	Photoelectrochemical water splitting	[250]
TiO_2_	CNCs	titanium(IV) ethoxide	Sorbent for microextraction	[264]
TiO_2_	CNCs	Ti-n-butoxide	Photocatalyst–(degradation of methylene blue)	[265]
TiO_2_	CNCs	tetra-n-butyl titanium	Photocatalyst–degradation of Rhodamine B	[266]
α-Fe_2_O_3_	CF	FeCl_3_, calcination	Catalyst (oxidation of alcohols with H_2_O_2_)	[267]
α-Fe_2_O_3_	CF	FeCl_3_, calcination	Magnetic materials, catalyst	[268]
α-Fe_2_O_3_	CF	FeCl_3_, calcination	–	[269]
Fe_2_O_3_	RC	FeCl_2_, NaOH	–	[270]
Fe_3_O_4_ NPs (10 nm)	BNC	Fe(NO_3_)_3_, urea, hydrothermal reaction	Electrode in Li batteries	[271]
Fe_3_O_4_ NPs (10 nm)	BNC	Fe(NO_3_)_3_, urea, hydrothermal reaction	Flexible electrodes in Li batteries	[272]
Fe_2_O_3_ NPs	BNC	Fe(NO_3_)_3_, pyrolysis	Anode in Li-ion batteries	[273]
ZnO	CF	ZnCl_2_, NaOH, ethylenediamine	Photocatalyst (degradation of methyl orange)	[274]
ZnO	CF	Zn(NO_3_)_2_, urea, calcination	Photocatalyst (degradation of methyl orange)	[275]
ZnO (500–1000 nm)	MCC	Zn(NO_3_)_2_, NH_3_, microwaves, hydrothermal reaction	Photocatalyst (degr. of methylene blue and rhodamine B)	[276]
In_2_O_3_–SnO_2_ nanotubular (ITO)	CF	In(III) acetylacetonate, Sn(IV) isopropoxide	Active multilayers in photoanode	[277]
Co_3_O_4_ NPs (50–100 nm)	RC	CoCl_2_, calcination	Electrode in Li batteries	[278]
MnO_2_ NPs	MCC	Mn(CH_3_COO)_2_, CO(NH_2_)_2_, KOH, (CH_2_)_6_N_4_, NaOH, calcin	–	[279]
Co_0.5_Cu_0.5_Fe_2_O_4_	CF	Cu(II) acetate, Co(II) acetate, Fe(NO_3_)_3_, sol-gel process, calcination	–	[280]
V_2_O_5_, V_2_O_3_, Fe_3_O_4_, WO_3_ (200–1000 nm)	CF	Vanadium(V) oxychloride, tungsten(VI) chloride, iron(III) chloride	–	[281]
CoFe_2_O_4_ NPs (5–10 nm)	BNC	Fe(NO_3_)_3_, Co(NO_3_)_2_, NH_4_OH	Catalyst in fuel cells	[282]
MnO_2_ NPs	BNC	KMnO_4_, K_2_SO_4_, reduction by C	Electrode in supercapacitors	[283]
MnFe_2_O_4_	CF	Previously synthesized MnFe_2_O_4_	Adsorbent for As(III) and As(V)	[284]
LaNiO_3_	MCC	La nitrate, Ni nitrate, NH_3_, EDTA	Catalyst (steam CO_2_ reforming of methane)	[285]
SnO_2_	CF	(Sn(OiPr)_4_, sol-gel process, calcination	Electrode Li batteries	[286]
SnO_2_	BNC	SnCl_2_, pyrolysis	Anode in Li-ion batteries	[287]
**Inorganic Nanostructures Supported by Carbon Fibers Formed from Cellulose**				
Ge	BNC	GeBr_2_, pyrolysis	Anode in Li-ion batteries	[287]
Pd-Al	CF	Pd(NO_3_)_2_, Al(NO_3_)_3_ (decomposition in a reducing atm. H_2_/N_2_)	Catalyst–(hydrogenation of cyclohexene)	[288]
Ni	MCC	Ni(OOCCH_3_)_2_,	Catalyst (conv. of p-nitrophenol into p-aminophenol)	[289]
Ni/NiO NPs (2–100 nm)	CNCs	Ni(NO_3_)_2_, glycine, calcination	–	[290]
Ru NPs	BNC	RuCl_3_, pyrolysis	Electrode in Li–O_2_ batteries	[291]
Fe_3_C NPs (10–30 nm)	BNC	FeCl_3_, pyrolysis	Electrode in Li-ion or Na-ion batteries	[292]
MoS_2_ nanoleaves	BNC	Na_2_MoO_4_, CS(NH_2_)_2_, pyrolysis	Anode in Li-ion batteries	[293]
MoS_2_ NPs	BNC	(NH_4_)_6_Mo_7_O_24_, CH_4_N_2_S, hydrothermal process	Electrocatalyst in H_2_ evolution reaction	[294]
NiCo_2_S_4_	CF	Ni(NO_3_)_2_, Co(NO_3_)_2_, hexamethylenetetramine	Positive electrode in supercapacitors	[295]
BiOBr	CNFs	Bi(NO_3_)_3_, NaBr, ethylene glycol	Adsorbent for rhodamine B and Cr(VI)	[296]

**Table 5:** The list of publications on cellulose supported metal sulfide, hydroxyapatite, and other inorganic nanostructures. The list is organized from large cellulose structures (CF and RC) via the microstructured celluloses (MCC and MFC) to nanostructured celluloses (BNC and CNCs).

Inorg. Particle (Size—nm)	Cellulose Used	Precursor (Synthetic Path)	Application	Reference
**Cellulose Supported Metal Sulfides**				
CdS	CF	CdCl_2_, AgNO_3_, mercaptoacet. acid	Colorimetric paper test stripe for Hg	[307]
ZnS–MoS_2_ (200–1000 nm)	CF	Na_2_MoO_4_, ZnCl_2_, CH_4_N_2_S	Flexible Broadband Photodetector	[308]
AgInS_2_ NPs	CF	AgNO_3_, In(NO_3_)_3_, NaOH, cysteine	Nanodrug for hepatocellular carcinoma	[309]
CuS	MFC	CuSO_4_, Na_2_S, NaOH, NH_3_, xylan	Paper for ablation of pathogenic microorganisms	[310]
CuS (100–1000 nm)	BNC	CuSO_4_, Na_2_S	Electrode in supercapacitors	[311]
CdS	BNC	Cadmium(II) chloride, thiourea	Photocatalyst-methyl orange under UV	[312]
MoS_2_	CF	Na_2_MoO_4_, thiourea, MnCl_2_	Photocatalyst–degr. of RhB dye	[313]
ZnS	BNC	Zinc(II) acetate, thioacetamide	Fluorescent material	[314]
Zn_x_Cd_y_S	BNC	Cd(II) chloride, thiourea	Photocatalyst-H_2_ prod.-visible light	[315]
ZnO-CdS	BNC	Zinc(II) nitrate, Cd(II) sulfate	Photocatalyst–methyl orange–UV	[236]
MoS_2_ NPs (10 nm)	BNC	(NH_4_)_2_MoS_4_, N_2_H_4_	Electrocatalyst in H_2_ evolution react.	[316]
MoS_2_ NPs	BNC	(NH_4_)_2_MoO_4_, CH_4_N_2_S,	Electrode in Li-ion batteries	[317]
ZnS	CF	Zinc(II) nitrate hexahydrate, sodium sulfide	Photoluminescent material–security paper	[318]
In doped Mo(O,S)_2_	CNFs	MoCl_5_, InCl_3_, CH_3_CSNH_2_,NH_2_NH_2_	Photocatalyst for organic dyes	[319]
FeS NPs (10–20 nm)	CF	Iron(II) chloride, sodium sulfide	Uranium(VI) ions immobilization–water purification	[320]
Ni_3_S_2_ NPs	BNC	NiCl_2_, CN_2_H_4_S, NH_3_	Electrode in supercapacitor	[321]
**Cellulose supported hydroxyapatites**				
Ca hydroxyapatite	MFC	NaH_2_PO_4_, CaCl_2_	Adsorbent for Cr(VI) ions, H_2_O purification	[322]
Ca hydroxyapatite	BNC	NaH_2_PO_4_, CaCl_2_	Cell proliferation–bone tissue engin.	[323]
Ca hydroxyapatite	CF	Ca(NO_3_)_2_,CO(NH_2_)_2_ (NH_4_)_2_HPO_4_, NaOH,	Cell proliferation–bone tissue engin.	[324]
Ca phosphate–Ca hydroxyapatite	CF	CaCO_3_, P_2_O_5_, MgO Ca hydroxyapatite	Injectable bone paste	[325]
Ca hydroxyapatite	CF	Ca(NO_3_)_2_, (NH_4_)_2_HPO_4,_ NH_3_	Adsorbent for Hg, H_2_O purification	[326]
Ca hydroxyapatite	CNFs	Ca(NO_3_)_2_, (NH_4_)_2_HPO_4_	Fire resistant composite foams	[327]
Ca nanohydroxyapatite	CNCs	Commercial hydroxyapatite	Cell proliferation–bone tissue engin.	[328]
Ca hydroxyapatite	CNCs	Ca(NO_3_)_2_, (NH_4_)_2_HPO_4_	Adsorbent for chlortetracycline	[329]
**Cellulose supported miscellaneous inorganic nanostructures**				
CaCO_3_ NPs	CF	CaCl_2_, Na_2_CO_3_	Biomedical material	[330]
EuF_3_ NPs (250–600 nm)	CF	LiCl, EuF_3_	Phosphor–flexible optical material	[331]
Se NPs	CNCs	H_2_SeO_3_, Na_2_SeO_3_, sorbitol	-	[332]
Bi NPs (2–10 nm)	CNFs	Bi(NO_3_)_3_, NaBH_4_	Agent for enhanced radiation therapy	[333]
Ni(OH)_2_ NPs	BNC	NiSO_4_, NH_4_F, NH_3_	Electrode in supercapacitors	[334]
AgCl NPs	CF	AgNO_3_, NaCl	Antibacterial agent	[335]
AgCl/Ag (~1200 nm)	CF	AgNO_3_, AlCl_3_, NaOH	Photocatalyst, antibacterial agent	[336]
FeOOH	BNC	-	Flexible electrode-supercapacitors	[337]

**Table 6:** The list of publications on cellulose supported/templated inorganic hybrid nanostructures. The list is organized from large cellulose structures (CF and RC) via the microstructured celluloses (MCC and MFC) to nanostructured celluloses (BNC and CNCs).

Inorg. Particle (Size—nm)	Cellulose Used	Precursor (Synthetic Path)	Application	Reference
**Cellulose Supported Metallic Hybrid Nanostructures**				
Au (~100 nm)	CF	HAuCl_4_, cetyltrimethyl ammonium chloride, H_2_O_2_	Flexible electrode in environmental and biosensors	[357]
Au nanorods, Ag NPs, ZnO	CF	HAuCl_4_, H_2_NOH.HCl, ZnNO_3_, NH_3_, AgNO_3_, ethylene glycol	Electrode in electrochemical immunosensor	[358]
Au NPs (50–100 nm)	CF	HAuCl_4_, NaBH_4_, Na citrate, reduction	Flexible electrode sensor-oligonucleotides	[359]
Au NPs (30–100 nm)	CF	HAuCl_4_, trisodium citrate	Flexible plasmonic immunosensor for detection of sepsis	[360]
Au NPs, ZnO nanorods	CF	HAuCl_4_, cetyltrimethyl ammonium chloride, H_2_O_2_; Zn(NO_3_)_2_, (CH_2_)_6_N_4_,	Photoelectrochemical immunosensor-carcinoembryonic antigen antibodies	[361]
Au NPs, Mn_2_O_3_	CF	HAuCl_4_, ascorbic acid, Mn(NO_3_)_2_, glucose	Paper-based biosensor for Pb^2+^ detection	[362]
Au-Ag NPs	CF	HAuCl_4_, AgNO_3_, NH_3,_ ascorbic acid, reduction	Electrode in electrochemical immunosensor–carbohydrate antigen	[363]
Pt NPs (100–200 nm)	CF	H_2_PtCl_6_, ascorbic acid, reduction	Electrode in electrochemical immunodevice–tumor biomarker	[364]
Pt NPs–ZnO nanorods	CF	H_2_PtCl_6_, NaBH_4_, reduction; Zn(NO_3_)_2_, CH_3_COONH_4_, ethylenediamine	Electrode in H_2_O_2_ sensor–tumor cells detection	[365]
Pt NPs	CF	H_2_PtCl_6_, NaBH_4_, H_2_PtCl_6_, ascorbic acid, reduction;	Electrode in electrochemical sensor-DNA	[366]
Pt NPs	CF	H_2_PtCl_6_, ascorbic acid, reduction	Electrode in H_2_O_2_ sensor–cancer cells monitoring	[367]
Ag NPs	CF	AgNO_3_, ascorbic acid, reduction	Electrode in electrochemiluminescence sensor-antibodies	[368]
Ag NPs (20–100 nm)	CF	AgNO_3_, ascorbic acid, reduction	Electrode in immunodevice–cancer antigens	[369]
Ag NPs	CF	AgNO_3_, H_2_O_2_, NaOH, trisodium citrate	Self-healing superhydrophobicity	[370]
Pd-Au NPs (20–50 nm)	CF	Na_2_PdCl_4_ and HAuCl_4_, ascorbic acid, reduction	Electrode in H_2_O_2_ sensor–cancer cells monitoring	[371]
Au-Ag (100–500 nm)	CF	HAuCl_4_ and AgNO_3_, hydroxylammonium chloride, reduction	Electrode in cytodevice–glycan expression	[372]
Au-Pt (50–100 nm)	CF	HAuCl_4_ and H_2_PtCl_6_, ascorbic acid, reduction	Electrode in photoelectrochemical immunodevice-carcinoembryonic antigen	[373]
Pd-Au	CF	H_2_PdCl_4_ and HAuCl_4,_ ascorbic acid, reduction	Electrode in electrochemical immunodevice-carcinoembryonic antigen	[374]
Ag NPs	CF	Previously formed Ag NPs, AgNO_3_, Na citrate	Paper-Based SERS Sensor for Pesticide Detection	[375]
Ag NPs	CF	AgNO_3_, NaOH	Colorimetric assay for Hg^2+^ detection	[376]
Ag NPs	CF	AgNO_3_, dopamine hydrochloride	Antibacterial CA/TiO_2_/Ag NPs material	[377]
Au NPs (10–30 nm)	CF	HAuCl_4_, NaOH	Fluorescent probe for the detection of Hg(II) ions	[378]
Au-Pt NPs	CF	HAuCl_4_ and H_2_PtCl_6_, ascorbic acid, reduction	Electrode in cyto-device–H_2_S from cancer cells	[379]
Au NPs	CF	HAuCl_4_, NH_3_, trisodium citrate	Electrode in electrochemical biosensor for Pb^2+^ ions	[380]
Au NPs	CF	HAuCl_4_, cetyltrimethyl ammonium chloride, H_2_O_2_	Electrode in photoelectrochemical sensor-pentachlorophenol	[381]
Au NPs	BNC	-	Electrode in bioenzymatic sensor-glucose	[382]
**Cellulose Supported Metal Oxide or Sulfide Hybrid Nanostructures**				
ZnO platelet- superstructure	CF	Zn(NO_3_)_2_, trisodium citrate, NaOH	Electrode in photoelectrochemical immunodevice-carcinoembryonic antigen	[383]
ZnO nanorods	CF	Zn acetate, Zn(NO_3_)_2_, hexamethylenetetramine	Electrode in electrochemical immunosensor— α-fetoprotein	[384]
ZnO platelets, CuS NPs	CF	Zn(NO_3_)_2_, KCl, electrodeposition; Cu(NO_3_)_2_, NaS_2_	Electrode in photoelectrochemical (PEC) immunosensor-carcinoembryonic antigen	[385]
ZnO nanorods	CF	Pulsed laser deposition	Electrode in LED-photoelectrochemical immunoassay	[386]
SnO_2_ NPs	CF	SnCl_4_, graphene oxide	Electrode in chemiluminescence photoelectrochemical aptamer device	[387]
MnO_2_ nanowires	CF	MnSO_4_, Na_2_SO_4_, electrodeposition	Electrode in electrochemical immunosensor-prostate protein antigen	[388]
CdS NPs (~5 nm)	CF	CdCl_2_, Na_2_S, thioglycolic acid	Electrode in photoelectrochemical analytical device-adenosine triphosphate	[389]
Cu_1.3_Mn_1.7_O_4_ NPs (~50 nm)	CF	Cu(NO_3_)_2_, Mn(NO_3_)_2_, sol-gel process	Li-ion batteries	[390]
MoO_3_ (100–1000 nm)	CF	Previously prepared MoO_3_, (NH_4_)_6_Mo_7_O_24_, calcination	Colorimetric analysis system for Fe(III) ions	[391]
CoS NPs	BNC	Co(NO_3_)_2_, Na_2_S	Flexible electrodes-supercapacitors	[392]
FeOOH (5–50 nm)	CNCs	FeCl_3_, NaOH	Water defluoridation	[393]
ZnO-Ag	CNCs	Zn(OOCCH_3_)_2_, AgNO_3_, NaOH	Antibacterial agent	[394]
SnO_2_-Ag	CF	(Sn(OiPr)_4_, AgNO_3_, NH_3_, NaOH	Anodic material in Li-ion batteries	[395]
Co(Ac)_2–_Fe_3_O_4_	CNF	FeCl_3_, NH_3_, Co acetate	Nanocatalyst for 4H-pyrane and pyranopyrazole	[396]
TiO_2_ nanorods–Au NPs	CNCs	TiCl_4_, Ti(OBu)_4_, HCl, Au(Ac)_3_, 1-octadecene	Photocatalyst for degr. of Rhodamine B	[397]
AgNW-Fe_3_O_4_	CNF	AgNO_3_, ethylene glycol, PVP, FeCl_3_, FeCl_2_, NaOH	Electromagnetic interference shielding	[398]

## Data Availability

No new data were created or analyzed in this study. Data sharing is not applicable to this article.
